# Synthesis and Single Crystal X-ray Diffraction Structure of an Indium Arsenide Nanocluster

**DOI:** 10.1021/acscentsci.3c01451

**Published:** 2024-02-28

**Authors:** Soren F. Sandeno, Sebastian M. Krajewski, Ryan A. Beck, Werner Kaminsky, Xiaosong Li, Brandi M. Cossairt

**Affiliations:** Department of Chemistry, University of Washington, Box 351700, Seattle, Washington 98195-1700, United States

## Abstract

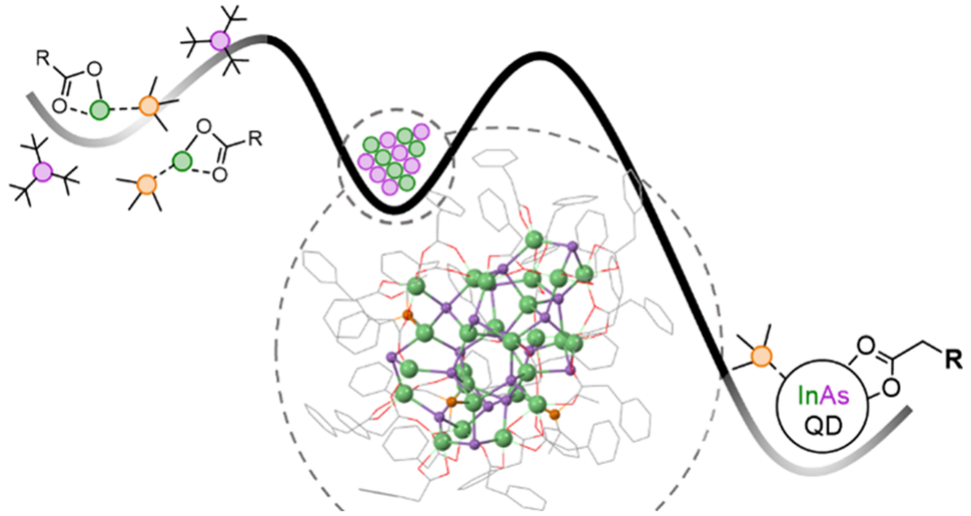

The discovery of magic-sized clusters as intermediates in the synthesis of colloidal quantum dots has allowed for insight into formation pathways and provided atomically precise molecular platforms for studying the structure and surface chemistry of those materials. The synthesis of monodisperse InAs quantum dots has been developed through the use of indium carboxylate and As(SiMe_3_)_3_ as precursors and documented to proceed through the formation of magic-sized intermediates. Herein, we report the synthesis, isolation, and single-crystal X-ray diffraction structure of an InAs nanocluster that is ubiquitous across reports of InAs quantum dot synthesis. The structure, In_26_As_18_(O_2_CR)_24_(PR'_3_)_3_, differs substantially from previously reported semiconductor nanocluster structures even within the III–V family. However, it can be structurally linked to III–V and II–VI cluster structures through the anion sublattice. Further analysis using variable temperature absorbance spectroscopy and support from computation deepen our understanding of the reported structure and InAs nanomaterials as a whole.

## Abstract 2

Magic sized clusters are ubiquitous intermediates in quantum dot synthesis. We isolate and structurally characterize an InAs nanocluster with a composition of In_26_As_18_(O_2_CR)_24_(PR'_3_)_3_.

## Introduction

Since the initial discovery of quantum dots (QDs), the mechanism by which they nucleate and grow has been the subject of intense experimental and theoretical study.^1−9^ While some nanocrystal syntheses have been rationalized through a model of classical nucleation involving a temporally discrete nucleation event, followed by growth,^10,11^ further investigation has shown that many systems deviate in complex ways from this classical model.^12,13^ One of the most important departures from the classical model of nucleation and growth is the formation of magic-sized clusters during the early stages of precursor conversion.^14−17^

Magic-sized clusters are atomically precise molecules that form as intermediates during the transition from precursors to nanocrystals. Their documentation in the literature is pervasive across QD chemistries and has led to a deeper understanding of the complexities in nanocrystal growth mechanisms.^12,13,18−23^ The formation of these clusters as intermediates breaks the kinetic chain linking precursor reactivity to nanocrystal nucleation and growth, meaning precursor tuning cannot be invoked to control size, shape, or morphology.^12^ While this generates synthetic challenges in controlling these material systems, some of the most impressive, industrially relevant syntheses of QDs use precursors known to result in intermediate cluster formation.^24,25^ The link between magic-sized clusters and superb QD quality continues to be under investigation to provide an understanding of why these two features are inextricably linked.

To understand the pathways of cluster formation and conversion, syntheses have been developed to allow for the isolation and characterization of these intermediates.^12,22,26,27^ The study of cluster intermediates not only informs the mechanism by which the ensuing QDs form but also produces a molecular platform upon which postsynthetic chemistry can be studied, including ligand exchange, doping, conversion to QDs, and even cluster interconversions and self-assembly.^28−37^ Many of the isolated clusters have been characterized optically, but the premier characterization of these materials has come in the form of single-crystal X-ray diffraction (SCXRD), which can allow for complete structural determination. This method has been used extensively in the characterization of metallic nanoclusters to unveil a deep, refined understanding of intermediate structures, growth pathways, and surface chemistry in those systems.^38^ While the number of reported semiconductor clusters is not as substantial, crystallography has demonstrated unprecedented phases that are not observable in the bulk, precise surface analysis of ligand binding modes and stoichiometries, and a more nuanced insight into the differences between clusters made up of different semiconducting materials.^18,22,39,40^ With a tenacious approach to structural characterization, the QD community will benefit from the same insights that have allowed great progress in the field of metallic nanoclusters. Toward that end, the structures of clusters as QD intermediates have been determined for a wide variety of the most common semiconducting QD materials, such as CdSe, CdS, and InP.^18,22,39,40^ However, many other material systems have identified the presence of magic-sized clusters spectroscopically but still lack full structural characterization of those intermediates.^20,27,41−45^

One of the QD material systems known to form clusters but lacks structural characterization is InAs. The synthesis of high-quality InAs QDs has been reported and improved upon for more than 20 years, with much attention paid to the final monodispersity and less to the formation mechanisms.^20,26,41,42,46−51,53^ Most reported syntheses use indium carboxylate, a tertiary alkylphosphine, and a reactive silylarsine to nucleate and grow InAs QDs. In studying this precursor system, magic-sized clusters were observed as an intermediate with a characteristic absorption doublet with maxima at 425 and 460 nm.^20,41,42,51^ Even when investigating the usage of germylarsines to improve nanocrystal monodispersity, this characteristic cluster persists.^42^ Since the initial optical characterization of the InAs magic-sized cluster, this precursor system has continued to be the standard for synthesizing high-quality InAs QDs. However, the synthesis, isolation, and structural characterization of the cluster intermediate have remained elusive.

Herein, we report a synthesis of InAs magic-sized clusters that allows isolating the material with various carboxylate and phosphine ligands. Further characterization of the isolated material by SCXRD has shown the structure to be In_26_As_18_(O_2_CR)_24_(PR'_3_)_3_ with a mixed carboxylate and phosphine ligand environment on the surface and a relatively anisotropic core structure with pseudo-*C*_3_ symmetry that persists to the ligand shell. This represents the first complete structural characterization of an InAs magic-sized cluster and provides new insights into comparative structures and structural evolution of III–V and II–VI clusters.

## Results and Discussion

### Synthesis and Isolation

Previous syntheses of InAs magic-sized clusters have shown that there are three essential precursors to allow for cluster formation: indium carboxylate, tertiary alkylphosphine, and silylarsine.^20,41,51^ This is compared to InP cluster syntheses, which do not necessarily require an alkylphosphine for cluster formation or high-quality QD growth. The role of the tertiary alkylphosphine is understood to be 2-fold as it has been cited to function as a Lewis base to activate the indium carboxylate precursor as well as decrease the apparent reactivity of silylpnictides through solvation effects similar to amines.^42,51,52^ This suggests that the alkylphosphine requirement for InAs syntheses is likely a result of the higher reactivity of As(SiMe_3_)_3_ compared to P(SiMe_3_)_3_. The more reactive arsine precursor requires an increase in the reactivity of the indium carboxylate to achieve balanced reactivity and controlled nucleation. This can be accomplished by including the alkylphosphine to activate the indium while hindering the diffusion of the As(SiMe_3_)_3_.

**Figure 1:**
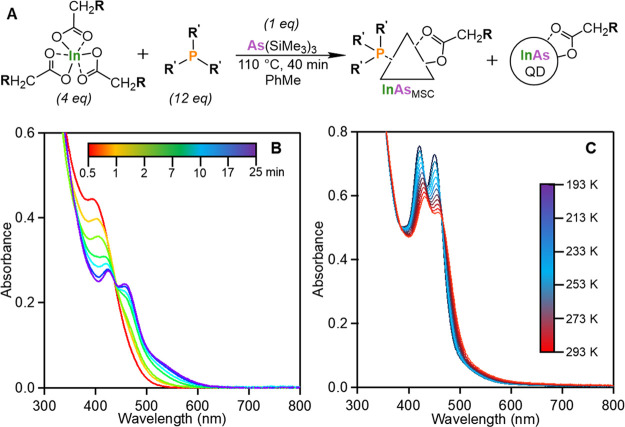
A) Reaction scheme for synthesizing alkyl carboxylate and tertiary phosphine ligated InAs clusters. B) Absorbance progression of a typical InAs cluster synthesis using indium myristate and trioctylphosphine. C) Variable temperature absorbance of InAs clusters from 20 °C (red) to −80 °C (dark blue) in 10 °C increments in toluene.

In a typical synthesis, the indium carboxylate is generated through the reaction between indium acetate and carboxylic acid under vacuum or using trimethylindium and carboxylic acid in solution. The phosphine is added to the indium precursor, and the temperature is raised to 110 °C. Once at the reaction temperature, a solution of As(SiMe_3_)_3_ is injected, immediately starting the reaction, as shown in Figure 1B. We have found that hot-injection of As(SiMe_3_)_3_ into just indium carboxylate at 110 °C results in no productive cluster or QD formation, but the injection of the phosphine into this mixture immediately initiates cluster formation (Figure S1). This result corroborates the findings on the role of the phosphine mentioned above and implicates the phosphine as necessary for balancing the precursor consumption rates that promote kinetic trapping and cluster formation. As clusters evolve at 110 °C, a substantial amount of QD forms as a side-product. The relative stoichiometry of phosphine appears to have an effect on the yield of the cluster with respect to the amount of unproductive QD growth. There is a noticeable increase in undesirable QD formation when using myristate ligands outside the range of one and three equivalents of phosphine relative to indium. We propose that if the amount of phosphine is too low, not enough of the indium precursor is activated, leading to As(SiMe_3_)_3_ reacting with lower equivalents of activated indium. If the amount of phosphine approaches and exceeds three equivalents, the equilibrium is pushed toward a different surface ligand stoichiometry as the cluster forms, causing destabilization and QD growth.

While investigating ligand rigidity on the cluster surface to promote single-crystal growth, we have concluded that the carboxylate chain is also crucial in directing cluster formation. The myristate-based indium precursor with a long-chain hydrocarbon tail promotes the best control, yield, and robustness of the InAs cluster reaction. When transitioning to phenylacetate, we noted that the equivalents of organophosphine with respect to indium must be modified. The one to three equivalent window is no longer appropriate for indium phenylacetate, and instead, we find that not only does one equivalent of phosphine promote the best cluster growth, but three equivalents of phosphine results in no cluster evolution. This difference in phosphine requirement between carboxylates is likely a function of the phosphine-bound indium carboxylate precursor speciation, which has been found to vary with phosphine concentration.^51,54^ We, therefore, suggest that while rigid ligands such as phenylacetate allow for structural determination through single-crystal growth as described below, the most robust InAs cluster syntheses are those that are heavily modulated by hindering the diffusion of As(SiMe_3_)_3_ using long chain fatty acids.

Further synthetic modifications to allow for single-crystal growth involved the use of diethylphenylphosphine (Et_2_PhP) in place of *n*-alkylphosphines. The yield of the InAs cluster decreased substantially when using Et_2_PhP, as evidenced by an increase in the QD absorbance and a decrease in the peak-to-trough ratio of the cluster in the crude reaction. We observe no significant synthetic differences in the InAs cluster when using tri-*n*-octylphosphine or tri-*n*-butylphosphine, which suggests that there is not an appreciable change in diffusion rate as modulated by the phosphine (Figure S2). Furthermore, the cone angle of the *n*-alkylphosphines (∼132°) is quite similar to that of the Et_2_PhP (136°). The electronic parameters reported for these phosphines are different, however, with tri-*n*-butylphosphine reported as 2060.3 cm^–1^ and Et_2_PhP as 2063.7 cm^–1^.^55^ We suggest then that the binding affinity of the phosphine plays an important role in regulating the reactivity.

With the absorbance progression of the InAs cluster synthesis shown in Figure 1B, we also document the formation of an initial absorbance feature at 395 nm within the first 20 s of As(SiMe_3_)_3_ injection. This then converts through an isosbestic point, giving way to the 425 and 460 nm features characteristic of the final InAs-460 cluster. The rate of conversion of InAs-395 to InAs-460 is concentration-dependent, which would suggest a conversion process based on growth as opposed to a structural rearrangement (Figure S3). It would then seem that the InAs-395 is smaller compared to InAs-460 based on the energy of the first excitonic feature. Despite the concentration dependence of the cluster conversion rate, we document no significant difference in the overall yield of the InAs cluster or the cluster-to-QD ratio when varying total reaction concentration (Figure S4).

Purification of the InAs-460 cluster by size-exclusion chromatography allowed for further optical characterization through variable temperature absorbance measurements. From 20 °C to −80 °C, we observe significant spectral line narrowing consistent with vibronic coupling (Figure 1C). The narrowing, along with no absorbance changes upon returning to 20 °C, is consistent with previous variable temperature measurements on III–V cluster materials.^18,56^ Along similar lines, the isolated cluster shows no measurable photoluminescence at room temperature and, when cooled to 77 K, shows broad emission ranging from 550 nm to beyond 700 nm (Figure S5). This is not unexpected as the relatively low effective electron mass in bulk InAs (m_e_ = 0.023)^57^ should allow for many nonradiative recombination pathways through exciton-surface interactions. The narrow absorbance features at room temperature for quantum-confined InAs combined with the high degree of monodispersity as evidenced by spontaneous superlattice formation upon preparation of films for transmission electron microscopy analysis (Figure S6) suggested atomic precision, motivating our pursuit of diffraction quality crystals of InAs-460 for single crystal X-ray analysis.

### Crystal Growth and Structural Analysis

**Figure 2:**
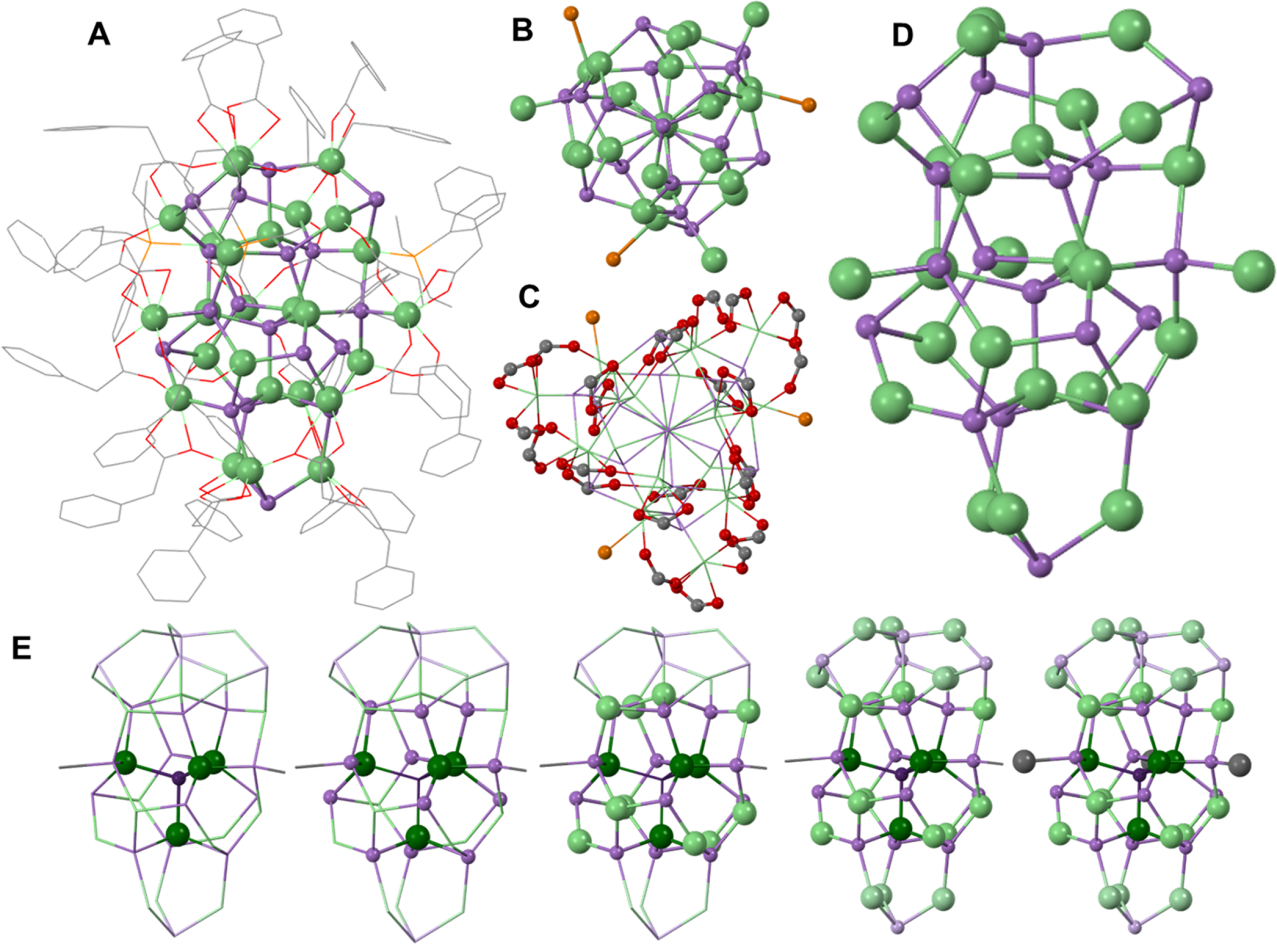
A) Full In_26_As_18_(O_2_CR)_24_(PR'_3_)_3_ cluster structure with ligands shown as a wireframe. B) View of In_26_As_18_ down the *C*_3_ axis showing the symmetry and propeller shape generated by the three bound P atoms. C) View of In_26_As_18_ down the *C*_3_ axis showing the symmetry of the bound carboxylates, which have been truncated at the carbonyl carbon. The core In_26_As_18_ structure is shown as a wireframe. D) Inorganic core of In_26_As_18_ with ligands removed for clarity. E) Components of the In_26_As_18_ cluster that first generate the cage and then cap the cage to complete the structure. The atom color is lightened as the structure extends to emphasize the layers of the cluster. Color legend: indium (green), arsenic (purple), phosphorus (orange), oxygen (red), carbon (gray). Hydrogen atoms have been removed for clarity.

Modifying the synthesis of InAs clusters using indium phenylacetate and diethylphenylphosphine as ligands (Figures S7, S8) allowed for the growth of X-ray quality single crystals from vapor diffusion (Figure S9). Diffraction at a resolution of 0.86 Å with R_1_ and R_w_ values of 14.09% and 28.02%, respectively, allowed for a high-quality structural solution, providing the assignment In_26_As_18_(O_2_CCH_2_Ph)_24_(PEt_2_Ph)_3_ (Figure 2A). Full crystallographic details are provided in Table S1.

The surface of this In_26_As_18_ cluster is ligated by 24 carboxylates and three phosphines. The carboxylate ligands show a binding mode distribution of nine chelating, three symmetric bridging, and 12 asymmetric bridging carboxylates. This affinity for asymmetric bridging carboxylates is consistent with other III–V cluster structures but the InAs seems to show an augmented ratio of the chelating binding mode in comparison to these other structures.^18,40^ It is interesting to note that the L-type binding of water in those previously reported InP clusters forces bridging ligands into a monodentate binding mode. However, in the InAs cluster, the L-type binding of phosphines is to otherwise 3-coordinate indium atoms, none of which are simultaneously ligated by carboxylates (Figure S10). The 8:1 ratio of carboxylate to phosphine is also represented in the ^1^H NMR spectrum of the purified material, showing that any excess alkylphosphine is efficiently removed through the purification by size-exclusion chromatography (Figure S11). There are only three surface indium atoms, which form a propeller around the *C*_3_ axis of the cluster. This symmetry is further enforced by the surface phosphines, which form a similar, yet mirrored, propeller around the same axis (Figure 2B). The *C*_3_ symmetry axis separates the cluster into three quadrants, each of which is ligated by four asymmetric bridging, one symmetric bridging, and three chelating carboxylates showing that the external structure of surface ligation maintains the symmetry dictated by the core (Figure 2C).

Removal of the surface indium results in a nonstoichiometric core of [In_23_As_18_]^15+^, which stands as a stark comparison to the previously reported core stoichiometries [In_21_P_20_]^3+^ and [In_14_P_13_]^3+^ in InP.^18,40^ It becomes evident that the drastic change in core stoichiometry is driven by the abundant 3-coordinate As atoms that make up nearly half the number of As present in the structure. We have previously concluded that in InP, there is a 4-coordinate requirement for the pnictide in cluster materials that forces a cation-rich stoichiometry through a large number of surface In atoms, passivating the otherwise 3-coordinate P. We see here that this is not the case in InAs. The vast majority of In is incorporated into the core of the cluster, allowing for seven 3-coordinate As atoms. This can potentially explain the lack of photoluminescence in InAs materials, as 3-coordinate pnictides have been linked to the formation of hole traps in tetrahedral InP QDs.^58^ Therefore, 3-coordinate As on the surface may be pervasive and a strong contributing factor to the photoluminescence of this material lagging behind other III–V and II–VI materials. As mentioned above, the lighter effective electron mass in InAs allows for efficient exciton coupling with surface states, so the effect of underpassivated atoms, such as 3-coordinate As, is more substantial compared to other materials. While InAs is more covalent than InP with an ionicity of 0.36 compared to 0.42, this likely cannot explain the difference in pnictogen undercoordination.^59^ However, it is well-documented that the basicity of arsines falls below that of their phosphine counterparts due to more s-character in the hybridized orbital of the As lone pair.^60^ This would effectively decrease the favorability for surface indium coordination. The final category that the 3-coordinate As can inform is the relative surface coverage directed by carboxylate-ligated indium. The previously reported InP clusters that contain only 4-coordinate P atoms have, as a consequence, a cation-rich surface that is then completely passivated by carboxylates. This causes the ligand density at the surface to be extremely high. With the InAs cluster, the 3-coordinate As atoms drastically reduce the cation richness of the surface, and thus, there are significantly fewer carboxylates protecting the core of the structure (Figure S13). We then predict that diffusion to the cluster surface will be much faster in InAs than InP as the ligand barrier preventing diffusion of reactive species is much smaller. This is important information as we seek to adapt cluster-based QD syntheses using this InAs system.

The structure of this In_26_As_18_ cluster appears to be related to a motif previously observed in InP, CdSe, and CdS.^18,36,39,40,43^ We observe that our recently reported In_26_P_13_ structure can be superimposed onto the In_26_As_18_ structure, showing that the anion sublattice has a high degree of overlap (RMS = 0.323 Å), however the indium sublattice is substantially different (Figure S14). Observing the similarity in the pnictogen substructure leads us to hypothesize that the early stage InAs-395 intermediate likely has an As_13_ sublattice that matches both the shape and stoichiometry of the P_13_ sublattice of In_26_P_13_. Smaller atomically precise InAs clusters have been synthesized and structurally characterized previously by reacting InMe_3_ directly with tBuAsH_2,_ but the In_8_As_8_ core does not bear strong structural connections with the In_26_As_18_ lattice, making it an unlikely candidate for the InAs-395 intermediate (Figure S15).^61^

Interestingly, despite the strong similarities in the anion sublattices of the In_26_As_18_ and the In_26_P_13_ and In_37_P_20_ structures, there are also significant differences (Figure S16). Removing the surface indium atoms, we can compare the bond angles of the pseudo-wurtzite [In_21_P_20_]^3+^, [In_14_P_13_]^3+^, and [In_23_As_18_]^15+^ cores of the clusters to get a measure of the deviation in each cluster’s structure from the bulk wurtzite crystalline phase of the III–V materials. The E–In–E bond angle in bulk wurtzite is 109.5°, so a larger divergence from this value approximates a more strained structure. Measuring the E–In–E angles in the InP and InAs cluster cores show an average and standard deviation of 109.3° ± 5.6° for [In_21_P_20_]^3+^, 108.5° ± 6.3 for [In_14_P_13_]^3+^, and 113.8° ± 9.8 for [In_23_As_18_]^15+^. This shows that the core of the In_26_As_18_ cluster not only has an average that deviates the furthest from the bulk structure but also shows the widest variation in bond angles, as seen from the standard deviation. Direct comparison of this structure to the bulk wurtzite phase of InAs shows that the internal stack of alternating In_4_As and As_4_In tetrahedra in the structure follows a similar pattern to the bulk, but instead of eclipsing tetrahedra as in the bulk wurtzite phase, they are offset as the structure extends down the *c*-axis (Figures S17, S18).

To draw comparisons with the cage-like descriptions of previously reported clusters, there is a central, 4-coordinate As atom from which the cluster is built. Three 4-coordinate In atoms bind the central As to generate the cage framework. The cage is then closed by the external As atoms linked with ten additional In atoms. The cage is extended by adding four AsIn_3_ units, one of which crowns the cluster, and the other three are connected by the final As atom to generate the anisotropy (Figure 2D). The final cluster lattice is completed by adding three surface indium atoms that create the propeller. This progression is visually summarized in Figure 2E.

The inorganic core of the InAs cluster is similar to that of the Cd_26_Se_17_ structure formed through the cation exchange of Cu_26_Se_13_, which was recently reported by Zeng and co-workers.^36^ Comparison of the Cd_26_Se_17_ structure with In_26_As_18_ shows that the M_17_E_14_ cage structures and stoichiometries are nearly identical, including the anion and cation sublattices. That said, Cd_26_Se_17_ is almost a tetrahedron, whereas In_26_As_18_ is more anisotropic and bullet-shaped. This difference can be attributed to the attachment of three M_3_E units. In the case of Cd_26_Se_17_, there are three Cd_3_Se units that attach to the lower half of the Cd_17_Se_14_ cage to form the three corners of a tetrahedron. This attachment symmetry, combined with the Cd_3_Se unit that forms the top of the cage, creates a pseudotetrahedron shape. However, starting with a structurally homologous In_17_As_14_ cage, the three In_3_As units add even lower, to the bottom of the cage being linked together by the final, extra arsenic atom that pushes the stoichiometry to In_26_As_18_. This leads In_26_As_18_ toward the bullet-shaped structure (Figure S19).

### Computational Electronic Structure

**Figure 3:**
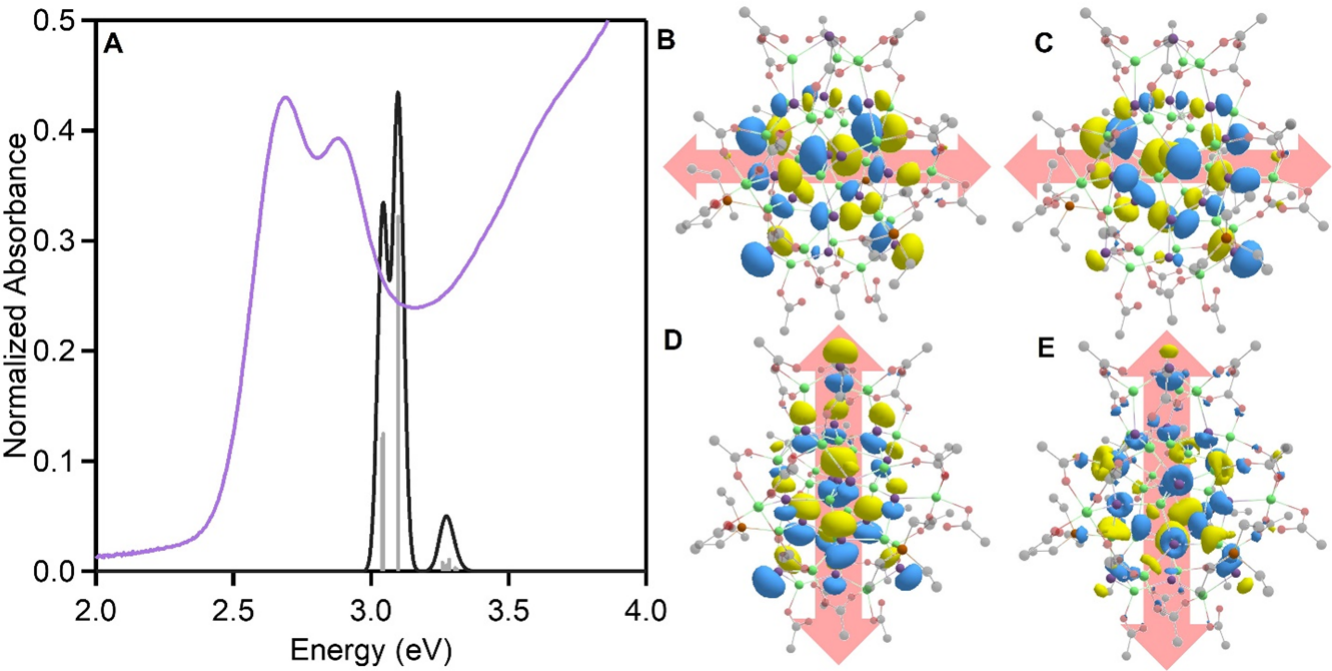
A) Experimental absorbance of InAs clusters with an applied correction for the Jacobian transformation (purple) compared to the calculated discrete absorbance states (gray) with a Gaussian broadening of 0.02 eV (black). B) Visualization of the HOMO leaving orbital oriented along the width of the cluster. C) Visualization of the HOMO–1 leaving orbital oriented along the width of the cluster. D) Visualization of the HOMO–2 leaving orbital oriented along the length of the cluster. E) Visualization of the LUMO arrival orbital for the first three transitions.

As a final point of characterization, we performed TDDFT calculations using the Gaussian software package,^62^ as detailed in the Supporting Information, to understand the orbital make-ups of the observed electronic transitions in the experimental absorption spectrum. The carboxylate ligands were truncated to acetate, and the phosphine ligands were modeled with ethyl groups to reduce computational costs (Figure S20). As is seen experimentally, there are two distinct absorption transitions; the first of which is comprised of both the HOMO and the HOMO–1, which are essentially degenerate, and the second consists of the HOMO–2 (Figure 3A). These leaving orbitals are primarily As 4p in character, with the most significant contributions from those closest to the cluster’s center. We note, however, that the HOMO and HOMO–1 orbitals are oriented across the width of the cluster (Figure 3B, 3C) as this contrasts with the HOMO–2 leaving orbital that participates in the second, higher energy transition, which is primarily oriented along the length of the cluster (Figure 3D). The arrival orbital for both transitions shows a significant degree of delocalization across the cluster structure and incorporates more In 5s character. There is, however, a qualitatively equal contribution of the As character to this arrival orbital, showing the high degree of covalency that is understood to occur in InAs (Figure 3E). Furthermore, with the isosurface contour plotted at 0.02, there is a small but noticeable contribution of p-character from the phosphine ligands to the arrival orbital distribution. This suggests that the ligand sphere, especially the phosphines, could be quite responsive to electronic excitation and may play a role in modulating the dynamics of the excited state. We note that the experimental absorption doublet is substantially red-shifted from the computationally predicted absorbance. However, the mismatch in energy is similar to that previously reported for comparable materials.^18,40^ Finally, the simulated Raman modes show good agreement with those characterized experimentally, falling in the range of 160–280 cm^–1^ (Figures S21, S22, S23). These energies are similar to those reported for the TO, SO, and LO modes of InAs nanowires at approximately 218, 237, and 239 cm^–1^, respectively.^63^

## Conclusions

In conclusion, we have presented the synthesis, isolation, and complete structural characterization of the first molecular InAs nanocluster. This species has been extensively documented spectroscopically by its characteristic absorption doublet at 425 and 460 nm as an intermediate in the synthesis of InAs QDs using indium carboxylate, alkylphosphine, and As(SiMe_3_)_3_. We report that phosphine concentration is important to the overall yield and purity of InAs-460 clusters, as too much or too little results in undesirable growth of InAs QDs as side-products. With long-chain aliphatic carboxylates and phosphines, we observe the presence of a second intermediate absorbing at 395 nm that converts through an isosbestic point into the In_26_As_18_ cluster. Diffraction-quality crystals of the isolated InAs-460 cluster were obtained using a combination of phenylacetate and diethylphenylphosphine. Single crystal X-ray diffraction revealed its composition as In_26_As_18_(O_2_CR)_24_(PR'_3_)_3_. While the overall atomic arrangement does not match any previously reported structures, the anion sublattice shares important similarities with previously reported InP and CdSe clusters. Computational simulation of the absorption spectrum and associated orbitals shows a high degree of delocalization, with the computed leaving orbitals being entirely composed of As p-character and the arriving orbital showing a distribution of electron density equally across In and As emphasizing the covalency of the InAs lattice. These findings are an important step in our understanding of the synthesis mechanisms in III–V QD systems and provide the structure of a molecular InAs cluster.

## Supporting Information Available

Complete experimental methods and supplementary data (NMR, Raman, FTIR, UV–vis, pXRD, TEM, crystallographic tables, and DFT calculations). Full crystallographic data can be found in the Cambridge Crystallographic Data Centre under deposition number 2308638. (PDF)
