# Supplementary material for: Synthesis and Single Crystal X-ray Diffraction Structure of an Indium Arsenide Nanocluster
Source: ACS Cent Sci. 2024 Feb 28;10(3):744–51. doi: 10.1021/acscentsci.3c01451 (PMC10979481; doi:10.1021/acscentsci.3c01451)
Supplement: Supplementary file 1 — oc3c01451_si_001.pdf [file oc3c01451_si_001.pdf]

Supplementary Information to Accompany:

Synthesis and Structure of an Indium Arsenide Nanocluster

Soren F. Sandeno, Sebastian M. Krajewski, Ryan A. Beck, Werner Kaminsky, Xiaosong Li, and Brandi M. Cossairt*

Department of Chemistry, University of Washington, Box 351700, Seattle, WA 98195-1700, United States

*cossairt@uw.edu

Experimental Methods.

General Considerations. Trioctylphosphine (97%), myristic acid (≥99%%), chlorotrimethylsilane (≥99%), phenylacetic acid (99%), tri-n-butylphosphine (≥93.5%), sodium metal (ACS reagent, dry), and naphthalene (99%) were obtained from Sigma Aldrich. Trioctylphosphine and tributylphosphine were vacuum distilled and naphthalene was sublimated prior to use. Dimethoxyethane (≥99%) obtained from TCI and distilled from sodium prior to use. Trimethyl indium (98%) and arsenic powder (99%) were obtained from Strem Chemicals and used as received. Diethylphenylphosphine (98%) was obtained from 1st Scientific and used without further purification. Deuterated benzene (C_6D_6) was obtained from Cambridge Isotope Laboratories, dried over CaH_2 and distilled prior to use. Tris(trimethylsilyl)arsine ($As(SiMe_3)_3$) was prepared according to a literature procedure for tris(trimethylsilyl)phosphine using metallic arsenic in the place of red phosphorus.[1] All manipulations were performed under an inert atmosphere of dry N_2 using standard Schlenk line or glovebox techniques unless otherwise indicated. Optical spectra were acquired on Cary 5000 and Cary 60 UV-Vis spectrophotometers from Agilent Technologies. NMR spectra were acquired on 300 and 500 MHz Bruker Avance spectrometers in C_6D_6. Diffractograms were collected on a Bruker D8 powder x-ray diffractometer with a Pilatus 100K large-area 2D detector using a 1 mm collimator. All solution phase FT-IR measurements were acquired on a PerkinElmer Frontier FT-IR spectrometer using a CaF_2 window liquid cell with the sample dissolved in tetrachloroethylene. The solvent signal was subtracted as a background before plotting. ICP-OES was conducted on a Perkin Elmer Optima 8300 inductively couple plasma – optical emission spectrophotometer after sample digestion overnight in 1:1 70% nitric acid/hydrogen peroxide and dilution to 2% nitric acid.

Synthesis of $In_{26}As_{18}(O_2C(CH_2)_{12}CH_3)_{24}(TOP)_3$. In a typical synthesis, myristic acid (0.548 g, 2.4 mmol) is added to an oven-dried 15 mL 3-neck round-bottom flask fitted with a thermowell, T-adapter, and septum. The flask is placed under vacuum at 50 mtorr for 30 minutes after which the system is placed under positive nitrogen flow and anhydrous toluene (2 mL) is injected into the flask. Trimethylindium (128 mg, 0.8 mmol) is dissolved in 1 mL of toluene and added dropwise to the flask with 800 rpm stirring at 2 drops per second. The solution is left to stir for 30 minutes after which trioctylphosphine (1.0 mL, 0.831 g, 2.24 mmol) is injected followed by stirring at 1200 rpm for an additional 15 minutes. The temperature is raised to 110 °C and within 5 minutes of reaching this temperature a solution of $As(SiMe_3)_3$ (60 uL, 0.2 mmol) in anhydrous toluene (0.5 mL) is then promptly injected into the flask. After 40 minutes at 110 °C, the reaction is allowed to cool to room temperature. Once cool, the crude reaction is purified directly by

size-exclusion chromatography in toluene in 0.75 mL increments. The toluene is removed under vacuum after purification to yield a dark orange, waxy solid.

Synthesis of $In_{26}As_{18}(O_2CCH_2Ph)_{24}(PEt_2Ph)_3$. Phenylacetic acid (0.654 g, 4.8 mmol) is added to an oven-dried 15 mL 3-neck round-bottom flask fitted with a thermowell, t-adapter, and septum. The flask is placed under vacuum at 50 mtorr for 30 minutes after which the system is placed under positive nitrogen flow and anhydrous toluene (2 mL) is injected into the flask. Trimethylindium (256 mg, 1.6 mmol) is dissolved in 1 mL of toluene and added dropwise to the flask with 800 rpm stirring at 2 drops per second. The solution is left to stir for 30 minutes after which diethylphenylphosphine (0.226 g, 279 uL, 1.6 mmol) is injected followed by stirring at 1200 rpm for an additional 15 minutes. The temperature is raised to 110 °C and within 5 minutes of reaching this temperature a solution of $As(SiMe_3)_3$ (120 uL, 0.4 mmol) in anhydrous toluene (0.5 mL) is then promptly injected into the flask. After 20 minutes at 110 °C, the reaction is allowed to cool to room temperature. Once cool, the crude reaction is purified directly by size-exclusion chromatography in toluene in 0.75 mL increments. The toluene is removed under vacuum after purification to yield a dark orange, crystalline solid. ICP-OES of the purified material showed an In:As ratio of 1.42:1. Elemental analysis $In_{26}As_{18}P_3O_{48}C_{222}H_{213}$: calculated C 33.0%, H 2.60%, N 0.00%; actual C 40.55%, H 3.64%, N 0.00%.

For crystallization, under a nitrogen atmosphere, 4 mg of isolated cluster is dissolved in 500 µL of toluene in a 2 mL screw top scintillation vial and the opening of the vial is completely covered with aluminum foil. This vessel is then sealed in a 20 mL scintillation vial with 3 mL of pentane. After 6 days of pentane diffusion into the toluene, bright orange crystals grew at the bottom of the inner vial. Single crystals of this material could also be grown using an identical method with diethyl ether in place of toluene.

Synthesis of $In_{26}As_{18}(O_2CCH_2Ph)_{24}(PBu_3)_3$. The procedure is the same as reported above for $In_{26}As_{18}(O_2CCH_2Ph)_{24}(PEt_2Ph)_3$ replacing diethylphenylphosphine with tri-n-butylphosphine (0.324 g, 395 uL, 1.6 mmol).

Computational Methods. Computational studies were performed using the Gaussian[2] electronic structure package. The HSE06[3–5] range-separated hybrid DFT functional was used to perform the linear-response TDDFT absorption spectra with the LANL2DZ basis set. This method has been used previously by our group to characterize the electronic structure of the $In_{37}P_{20}$ cluster.[6–8] Initial structures were taken from XRD, optimized until the total and root mean squared displacement and force were under $1.80*10^{-3}$ and $1.20*10^{-3}$ Bohr, and $4.50*10^{-4}$ and $3.00*10^{-4}$ Har/Bohr, respectively. Linear Response TDDFT[9–11] was used to compute both the excitation energies and corresponding oscillator strengths of the first 25 electronic transitions.

Single Crystal X-ray Diffraction Methods. An orange block from a toluene/pentane vapor diffusion, measuring 0.07 x 0.06 x 0.05 mm^3 was mounted on a loop with oil. Data was collected at -173°C on a Bruker APEX II single crystal X-ray diffractometer, Mo-radiation, equipped with a Miracol X-ray optical collimator. Crystal-to-detector distance was 40 mm and exposure time was 120 seconds per frame for all sets. The scan width was 0.7°. Data collection was 99.9% complete to 24.407° in Θ. A total of 176486 reflections were collected covering the indices, -44<=h<=44, -24<=k<=24, -43<=l<=43. 43904 reflections were symmetry independent and the elevated R_{int} = 0.2442 relates to the small sample size. Indexing and unit cell refinement indicated a monoclinic lattice. The space group was found to be P 2_1/c (No.14). The data was integrated and scaled using SAINT, SADABS within the APEX2 software package by Bruker.[12] Solution by direct methods (SHELXT[13]) produced a complete heavy atom phasing model consistent with the proposed structure. The structure was completed by difference Fourier synthesis with

SHELXL.[14,15] Scattering factors are from Waasmair and Kirfel.[16] Hydrogen atoms were placed in geometrically idealized positions and constrained to ride on their parent atoms with C---H distances in the range 0.95-1.00 Angstrom. Isotropic thermal parameters U_{eq} were fixed such that they were 1.2Ueq of their parent atom U_{eq} for CH's and 1.5Ueq of their parent atom U_{eq} in case of methyl groups. All non-hydrogen atoms were refined anisotropically by full-matrix least-squares. The contribution of disordered toluene and possibly pentane solvent to the diffraction pattern was removed with SQUEEZE,[17] and some disorder of the bound ligands was modeled. The crystallographic data for the structure has been deposited in the Cambridge Crystallographic Database under deposition number 2308638.

Supplemental Figures.

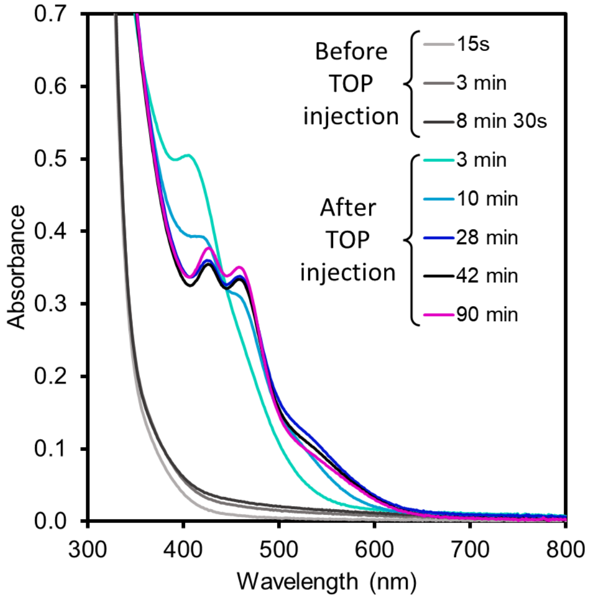

Figure S1. $As(SiMe_3)_3$ was injected into indium carboxylate in toluene as described in the synthesis of $In_{26}As_{18}(Myr)_{24}(TOP)_3$ in the absence of phosphine. With no development of recognizable absorbance features, 1.4 equivalents of trioctylphosphine with respect to indium was injected into the reaction causing the immediate formation of InAs clusters.

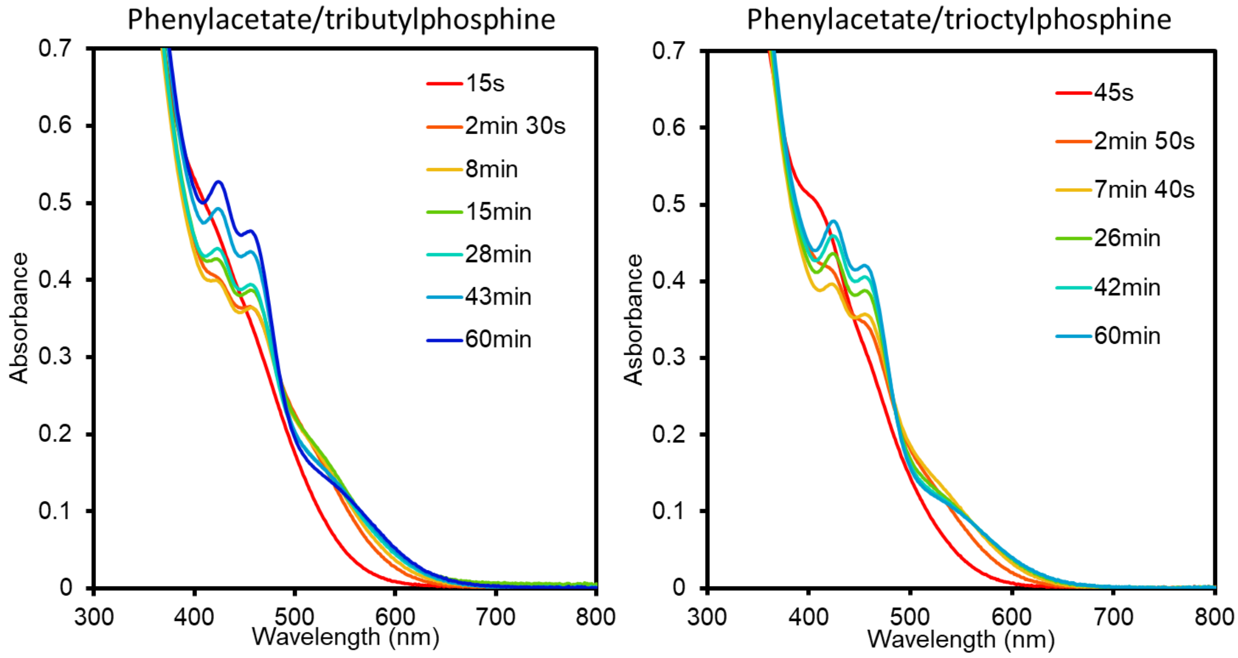

Figure S2. Equivalent InAs cluster reactions using two different phosphine ligands. The overall outcome of the synthesis as well as the kinetics of conversion do not seem to vary with n-alklyphosphine chain length.

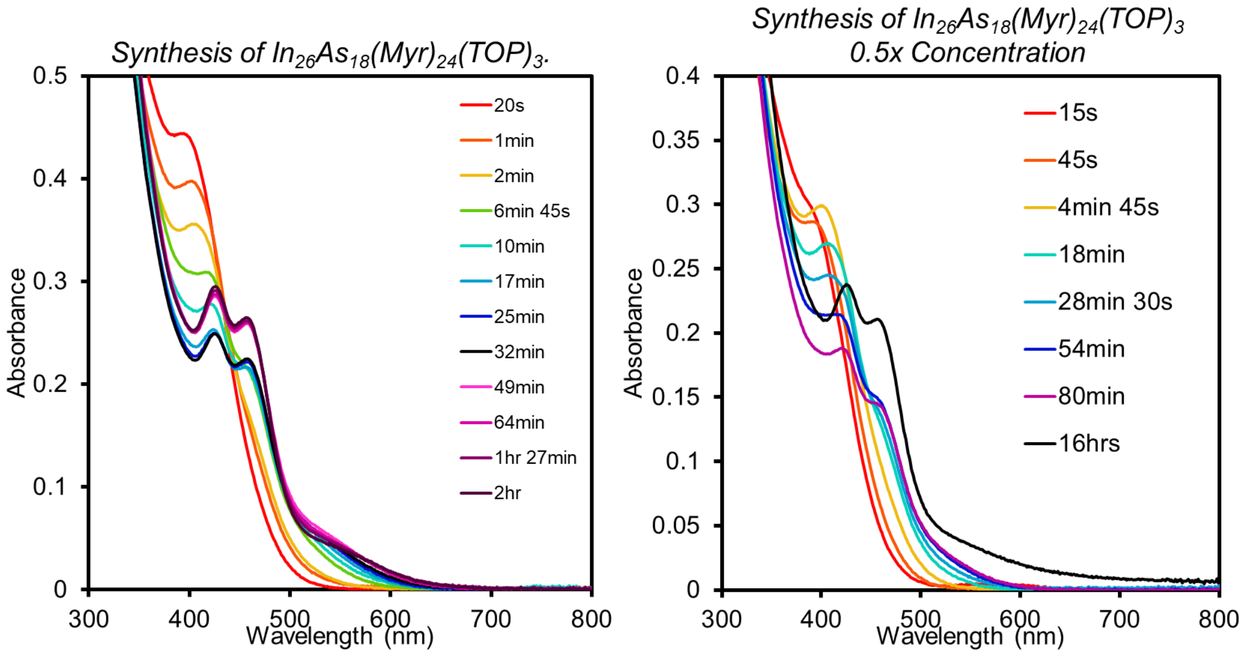

Figure S3. Absorbance progression for the synthesis of $In_{26}As_{18}(O_2C(CH_2)_{12}CH_3)_{24}(TOP)_3$ (left). Absorbance progression for the synthesis of $In_{26}As_{18}(O_2C(CH_2)_{12}CH_3)_{24}(TOP)_3$ with all reagents at half concentration in toluene (right).

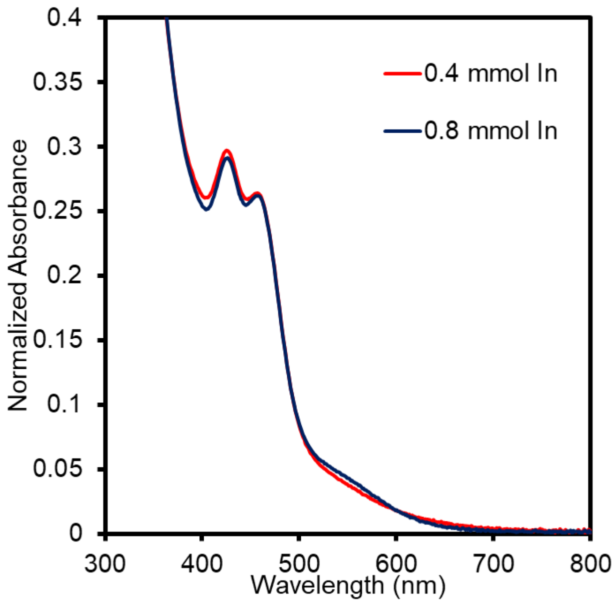

Figure S4. Final crude normalized absorbance traces from the reactions shown in Figure S3 suggesting the reaction outcome does not correlate meaningfully with concentration despite the stark differences in conversion kinetics.

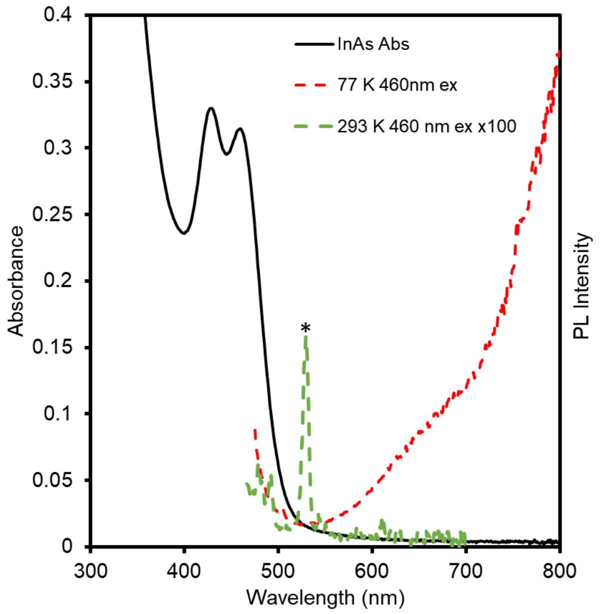

Figure S5. Photoluminescence of $In_{26}As_{18}(O_2CCH_2Ph)_{24}(PBu_3)_3$ at room temperature (green) and 77 K (red) with the absorbance shown in black. The sharp feature at 530 nm in the room temperature spectrum designated by the asterisk is a Raman feature from the solvent and does not represent photoluminescence from the sample.

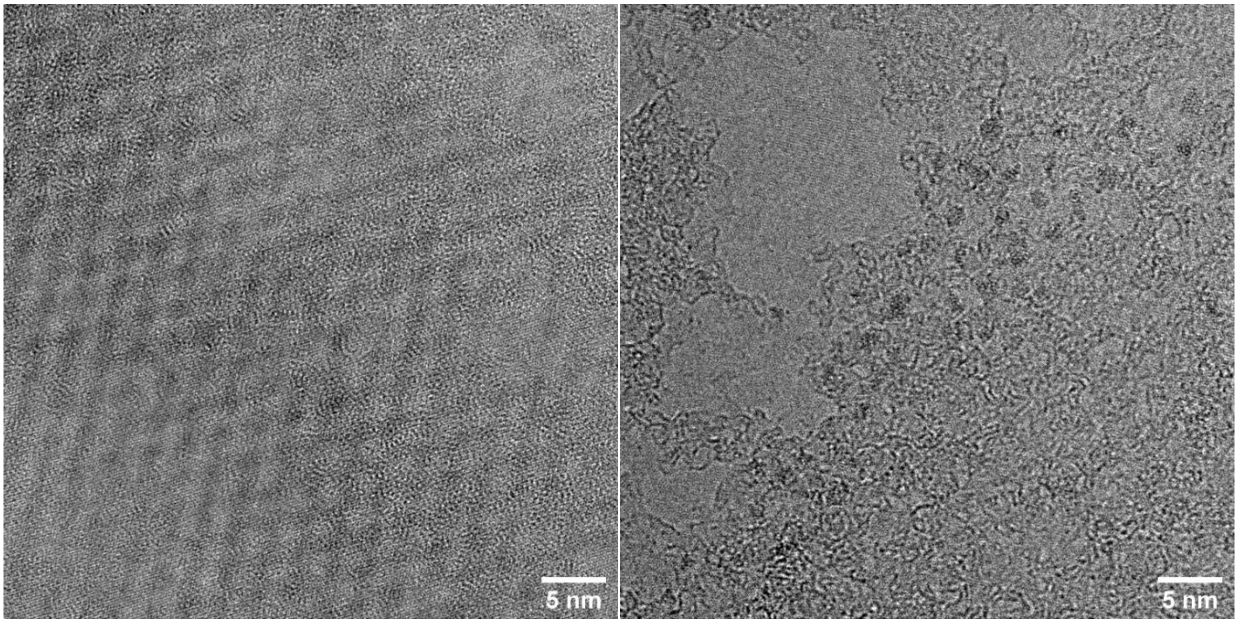

Figure S6. TEM micrographs of InAs clusters in a superlattice assembled by slow evaporation of toluene (left) and as discrete particles (right).

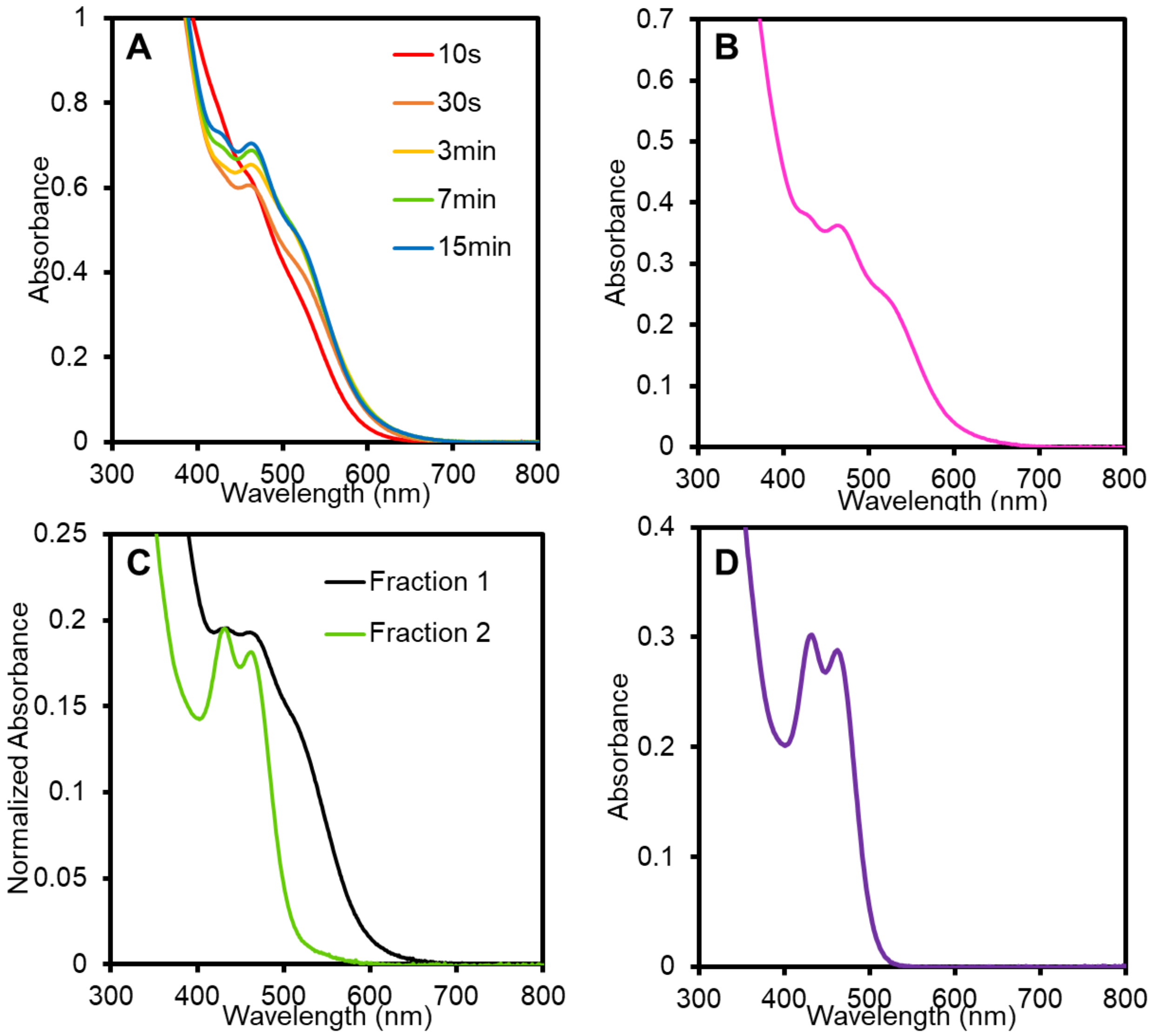

Figure S7. A) $In_{26}As_{18}(O_2CCH_2Ph)_{24}(PEt_2Ph)_3$ reaction progression showing that the overall cluster yield decreases when implementing the PEt_2Ph phosphine ligand. B) Final $In_{26}As_{18}(O_2CCH_2Ph)_{24}(PEt_2Ph)_3$ reaction trace after cooling and before purification. C) Absorbance traces of the two bands formed during size-exclusion chromatography. D) Absorbance of $In_{26}As_{18}(O_2CCH_2Ph)_{24}(PEt_2Ph)_3$ single crystals grown from an ether against pentane vapor diffusion and redispersed in toluene.

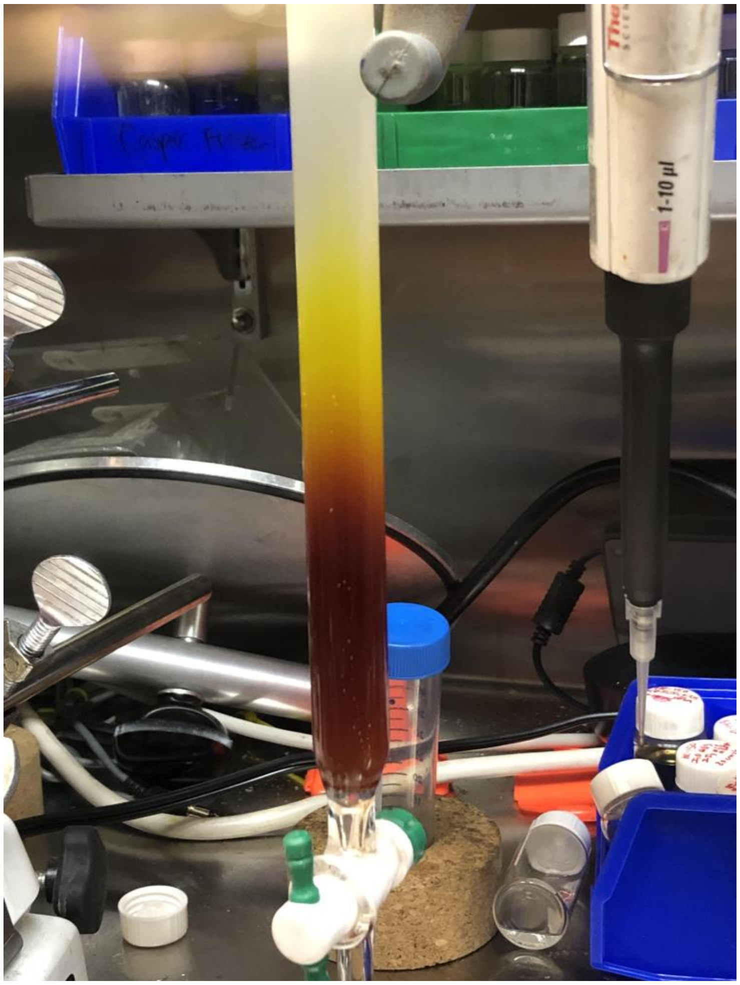

Figure S8. Direct purification of an InAs nanocluster reaction by size-exclusion chromatography. The yellow band is the $In_{26}As_{18}$ cluster and the dark red band is quantum dot impurity.

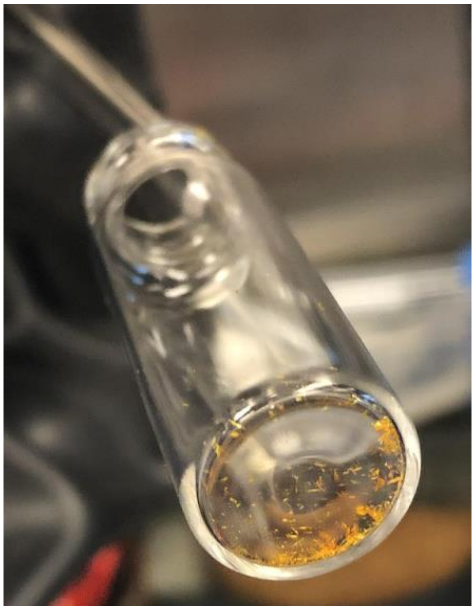

Figure S9. Photograph of the isolated single crystals grown from an ether against pentane vapor diffusion.

Table S1: Crystallographic data for the structures provided.

Empirical formula	C222 H213 As18 In26 O48 P3	
Formula weight	8075.70	
Temperature	100(2) K	
Wavelength	0.71073 Å	
Crystal system	Monoclinic	
Space group	$P\ 2_1/c$	
Unit cell dimensions	a = 36.969(3) Å	α= 90°.
	b = 20.6296(18) Å	β= 103.421(4)°.
	c = 36.014(4) Å	γ = 90°.
Volume	26716(4) $Å^3$	
Z	4	
Density (calculated)	2.008 Mg/m^3	
Absorption coefficient	4.489 mm^{-1}	
F(000)	15368	
Crystal size	0.070 x 0.060 x 0.050 mm^3	
Theta range for data collection	1.133 to 24.407°.	
Index ranges	-44<=h<=44, -24<=k<=24, -43<=l<=43	
Reflections collected	43904	
Independent reflections	43904 [R(int) = 0.2442]	
Completeness to theta = 24.407°	99.9 %	
Refinement method	Full-matrix least-squares on F^2	
Data / restraints / parameters	43904 / 10248 / 2775	
Goodness-of-fit on F^2	1.091	
Final R indices [I>2sigma(I)]	R1 = 0.1409, wR2 = 0.2368	
R indices (all data)	R1 = 0.3388, wR2 = 0.2802	
Largest diff. peak and hole	2.610 and -1.360 e.$Å^{-3}$	

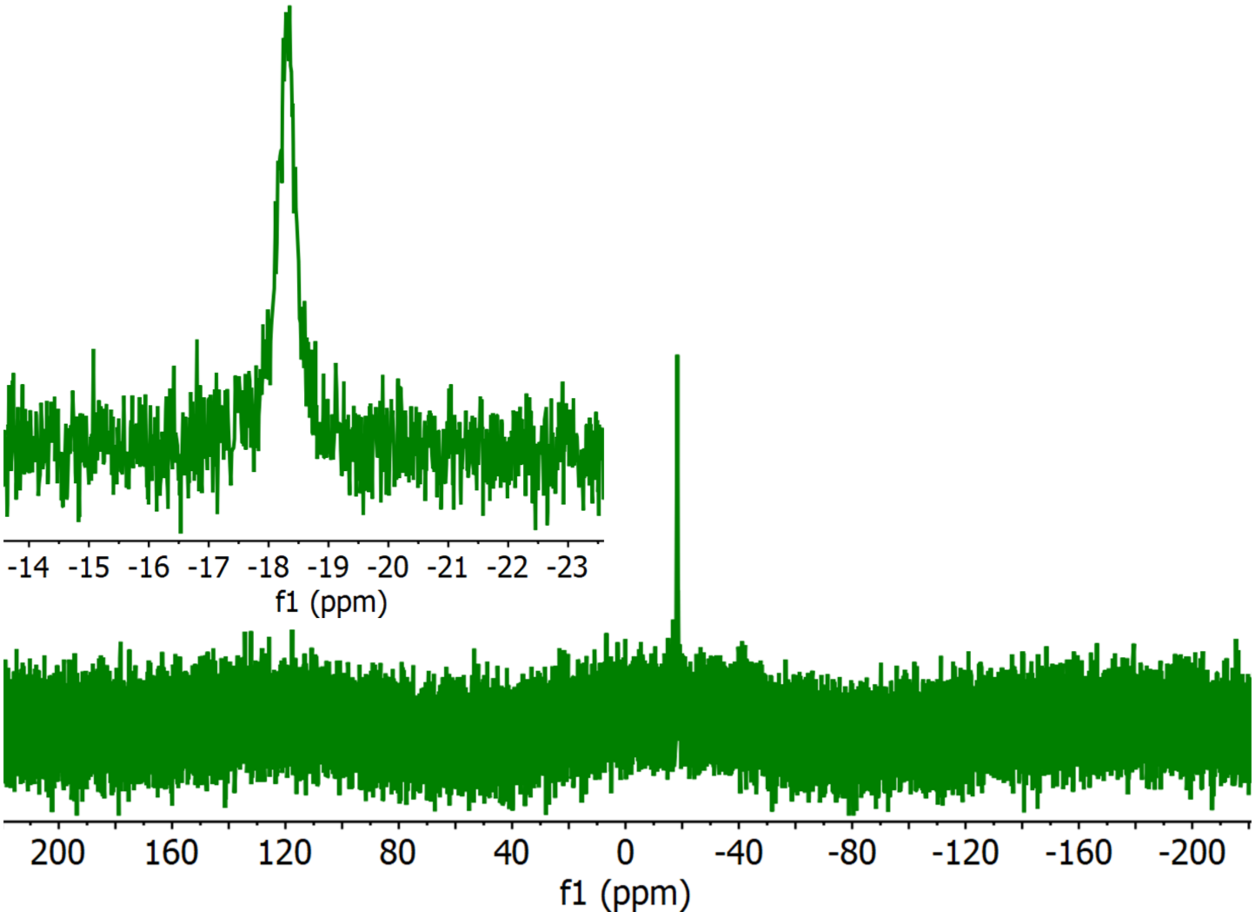

Figure S10. ^{31}P-NMR of purified $In_{26}As_{18}$ cluster with phenylacetate and tributylphosphine ligands. The inset focuses on the single feature at -18.4 ppm which is indicative of bound phosphine.

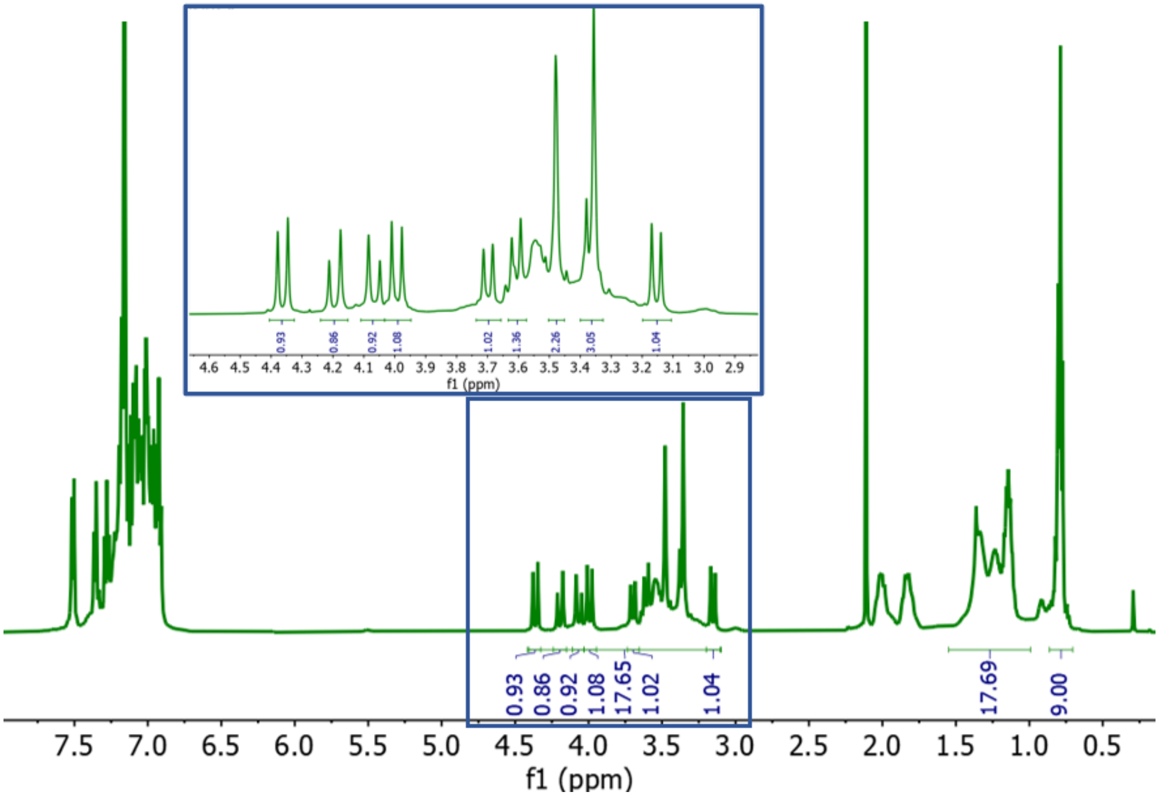

Figure S11. ^{1}H NMR spectrum of phenylacetate and tributylphosphine ligated InAs clusters. The inset shows the methylene region of the phenylacetate ligands.

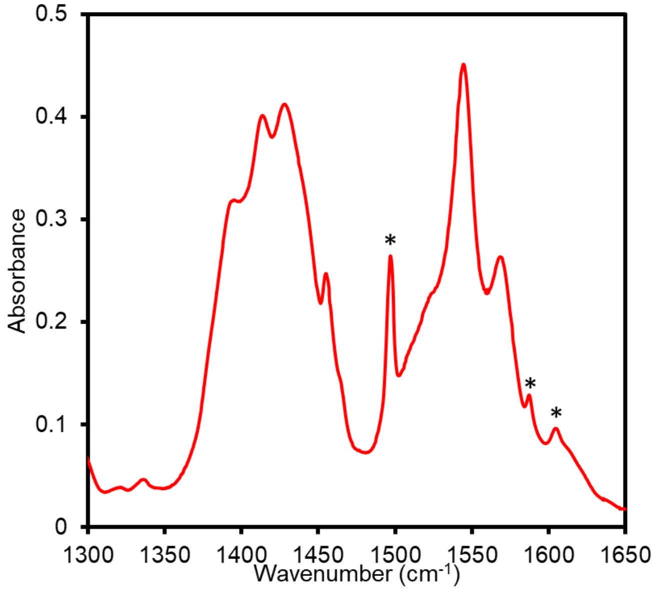

Figure S12. Solution-phase FTIR of phenylacetate and tributylphosphine ligated InAs clusters in tetrachloroethylene. The isolated ring breathing modes from phenylacetate are marked by asterisks.

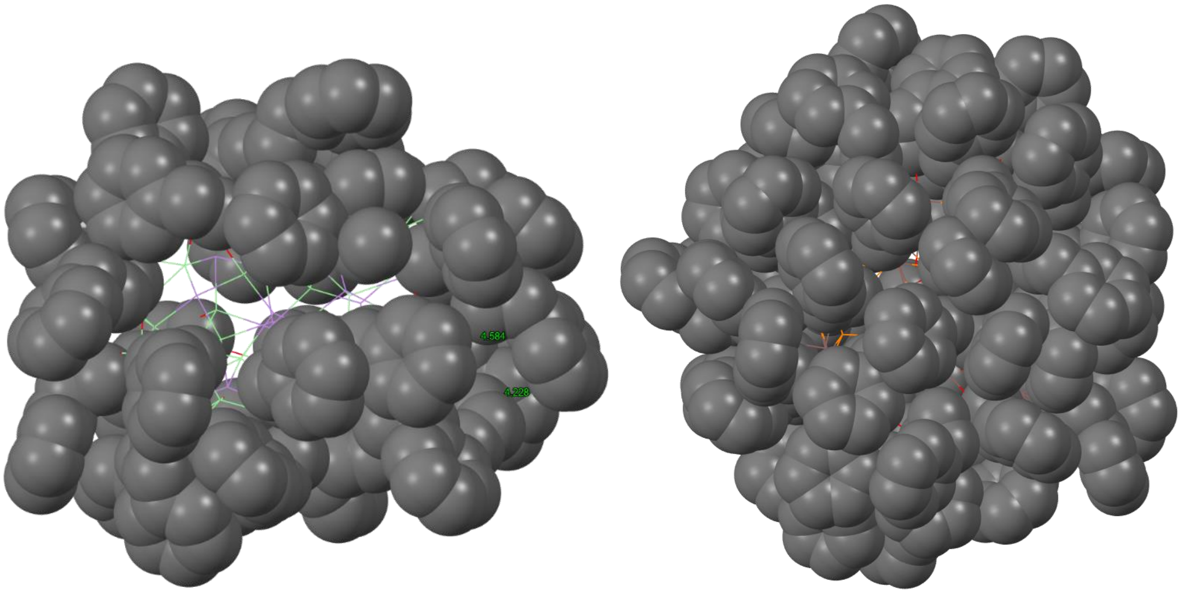

Figure S13. Fully ligated $In_{26}As_{18}(O_2CCH_2Ph)_{24}(PEt_2Ph)_3$ cluster (top). Fully ligated $In_{37}P_{20}(O_2CCH_2Ph)_{51}$ cluster (bottom). All carbons are presented through a space filling model and the underlying structures of the clusters are shown in wireframe. Hydrogens removed for clarity. 3-coordinate arsenic atoms that are present in the InAs cluster lead to less surface protection by carboxylates compared to the more cation-rich $In_{37}P_{20}$ cluster.

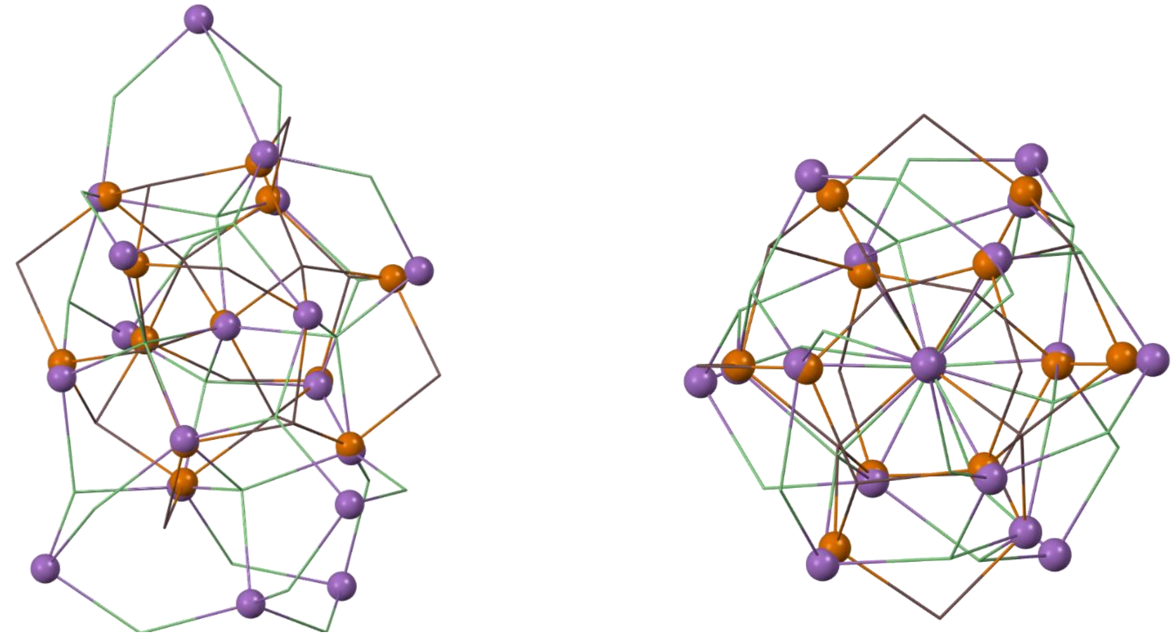

Figure S14. The overlay of the $In_{26}P_{13}$ and $In_{26}As_{18}$ anion sublattices. P (orange, ball), As (purple, ball), In from $In_{26}P_{13}$ (brown, stick), In from $In_{26}As_{18}$ (green, stick). RMS = 0.323 Å.

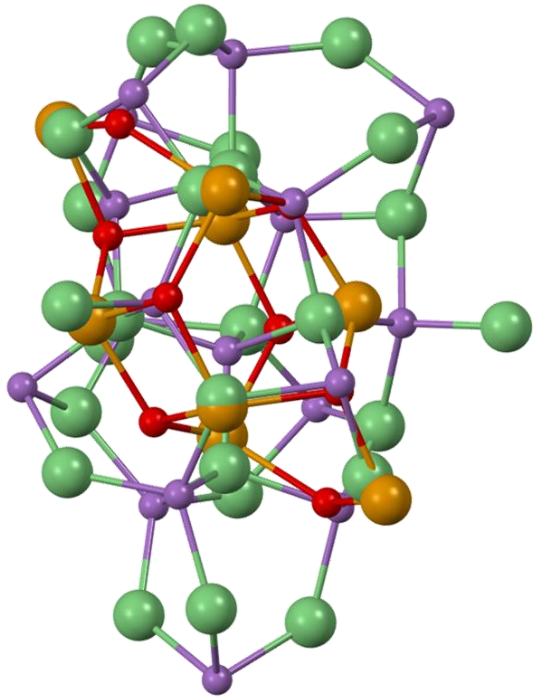

Figure S15. Structural overlay between $In_{26}As_{18}$ and In_8As_8. In from $In_{26}As_{18}$ (green), As from $In_{26}As_{18}$ (purple), In from In_8As_8 (gold), As from In_8As_8 (red).

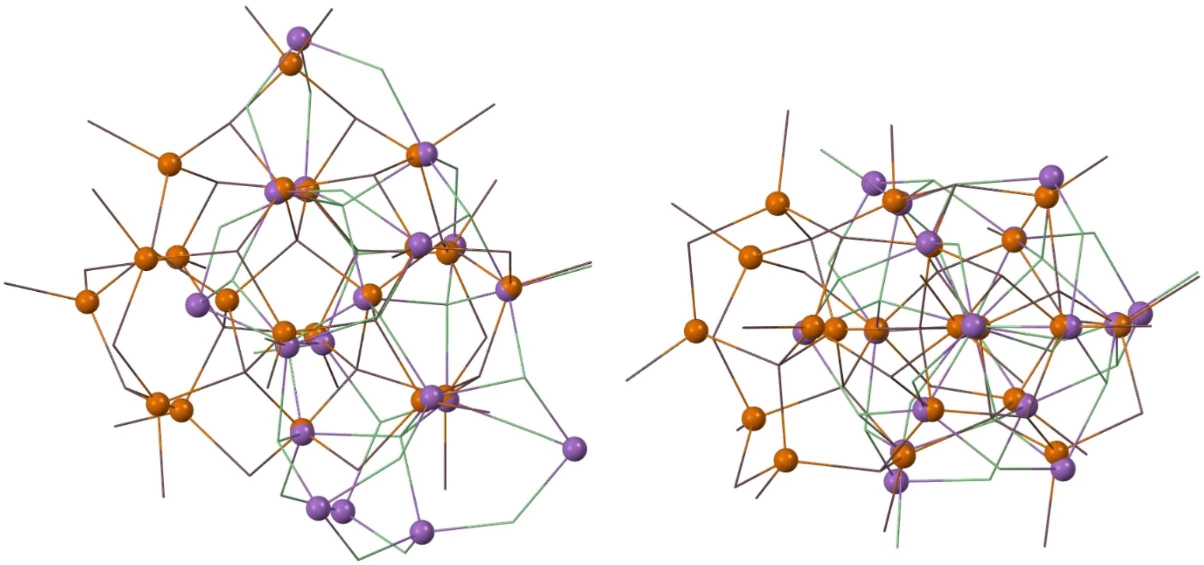

Figure S16. The overlay of $In_{37}P_{20}$ and $In_{26}As_{18}$ anion sublattices. P (orange, ball), As (purple, ball), In from $In_{37}P_{20}$ (brown, stick),.In from $In_{26}As_{18}$ (green, stick). RMS = 0.389 Å.

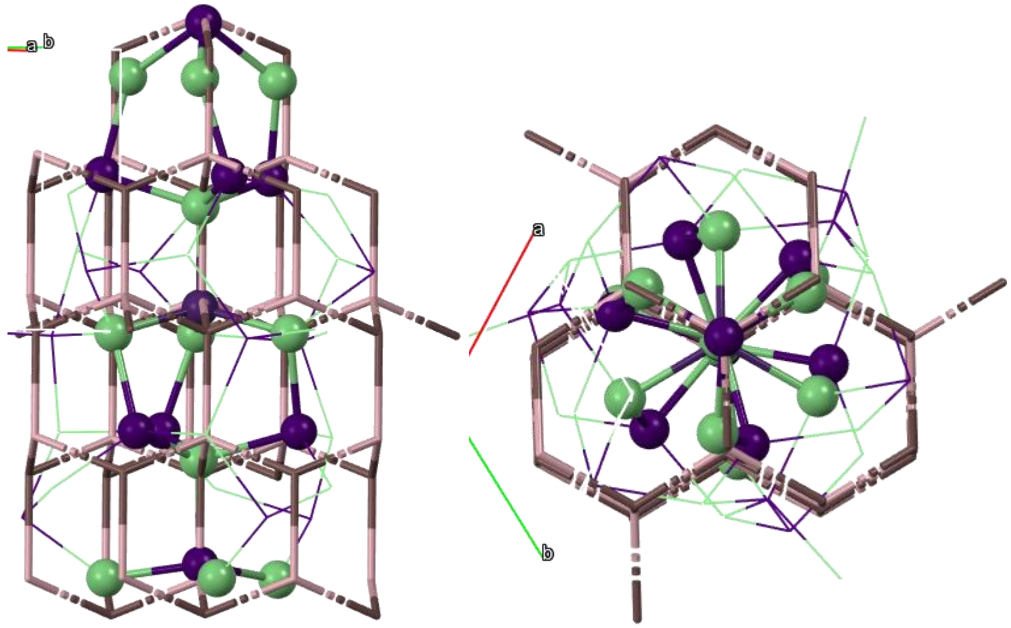

Figure S17. Structural overlap between the core $In_{26}As_{18}$ and bulk wurtzite InAs. The mirrored tetrahedra in the bulk wurtzite structure maintain an eclipsed relationship with no rotation as the lattice extends. With the pseudo-wurtzite phase of the cluster, the mirrored, alternating tetrahedra rotate slightly along the c axis misaligning the cluster phase with that of the bulk. For clarity, the core In and As-based tetrahedra of the cluster are shown in ball-and-stick whereas the atoms that extend beyond the central tower of tetrahedra are shown in wireframe. Color key: As from $In_{26}As_{18}$ (purple), In from $In_{26}As_{18}$ (green), As from bulk wurtzite InAs (pink), In from bulk wurtzite InAs (brown).

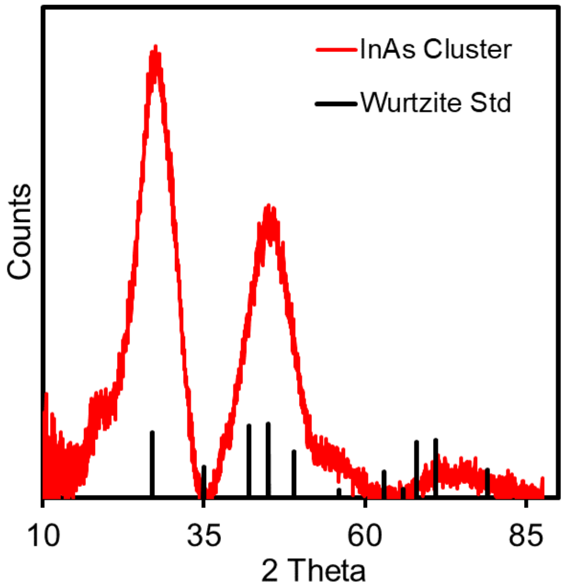

Figure S18. Powder x-ray diffraction of phenylacetate and tributylphosphine ligated InAs clusters (red). The diffraction standard of bulk wurtzite InAs is shows in black.

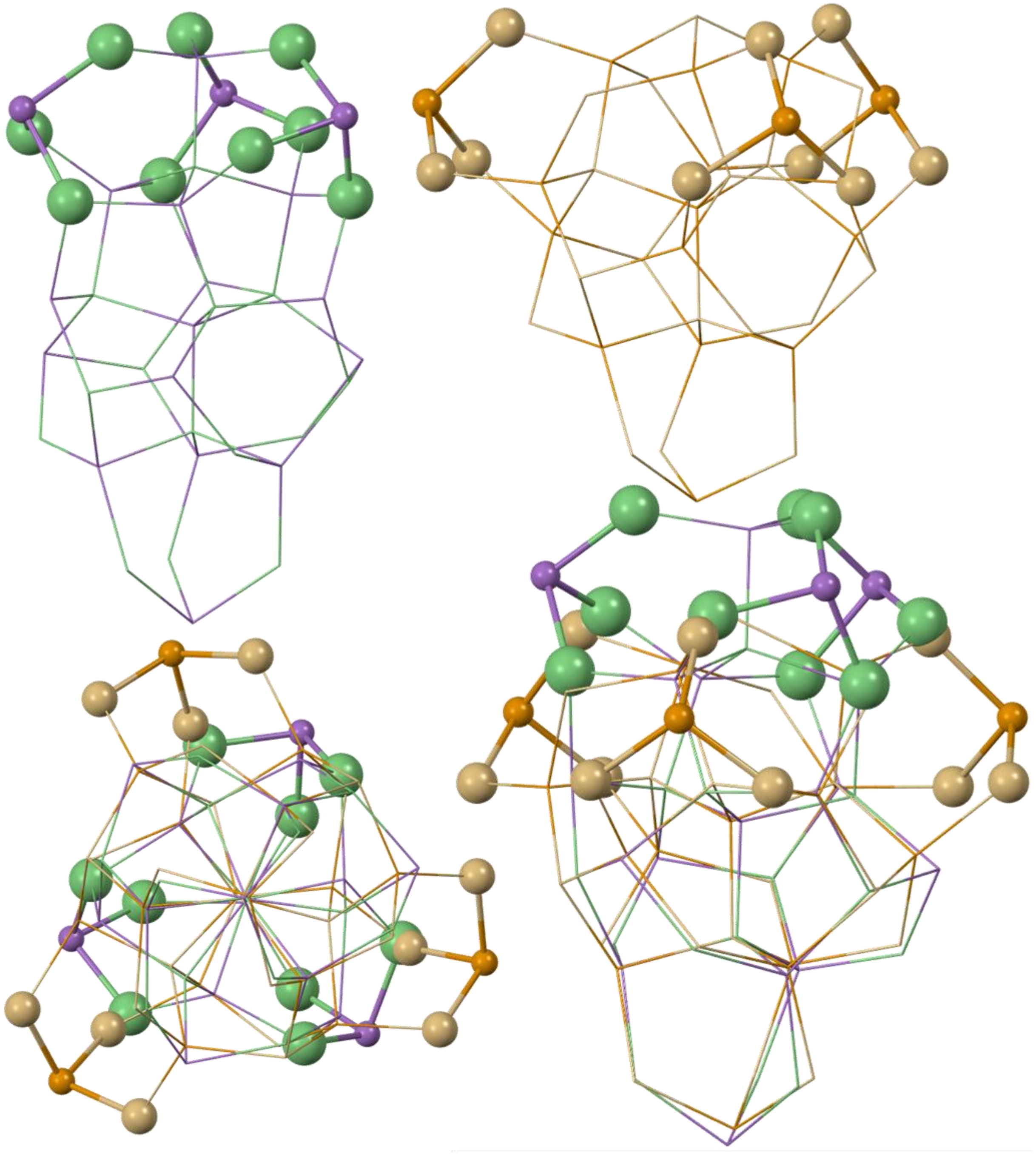

Figure S19. $In_{26}As_{18}$ structure with the surface In atoms removed shown in wireframe except for the three In_3As units that form the base of the bullet-like shape shown in ball-and-stick (top left). $Cd_{26}Se_{17}$ structure shown in wireframe except for the three Cd_3Se units that form the three corners of the base of the pseudo-tetrahedral shape shown in ball-and-stick (top right). Superimposed $Cd_{26}Se_{17}$ and $In_{26}As_{18}$ structures showing that the underlying $M_{17}E_{14}$ structure is homologous and the placement of the three M_3E units differentiate the two structures viewed from the side (bottom right) and down the C_3 axis (bottom left).

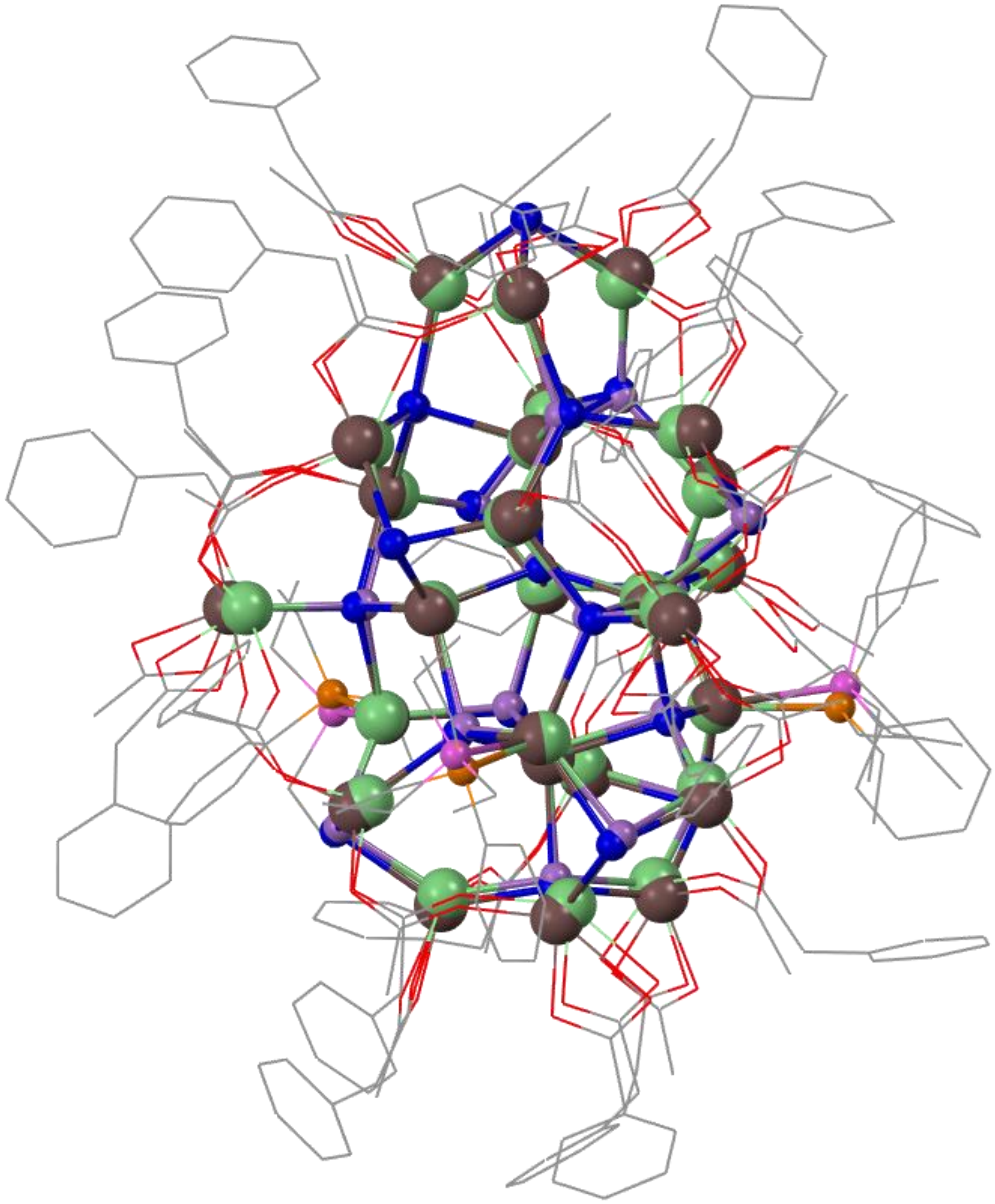

Figure S20. Structural overlay of experimentally determined x-ray structure and computed x-ray structure after geometric relaxation. The high degree of overlap indicates little geometric change for the computed structure. Color key: Experimental: As (purple), In (green), P (orange), O (red), C (grey) Computational: As (blue), In (brown), P (pink), O (red), C (grey).

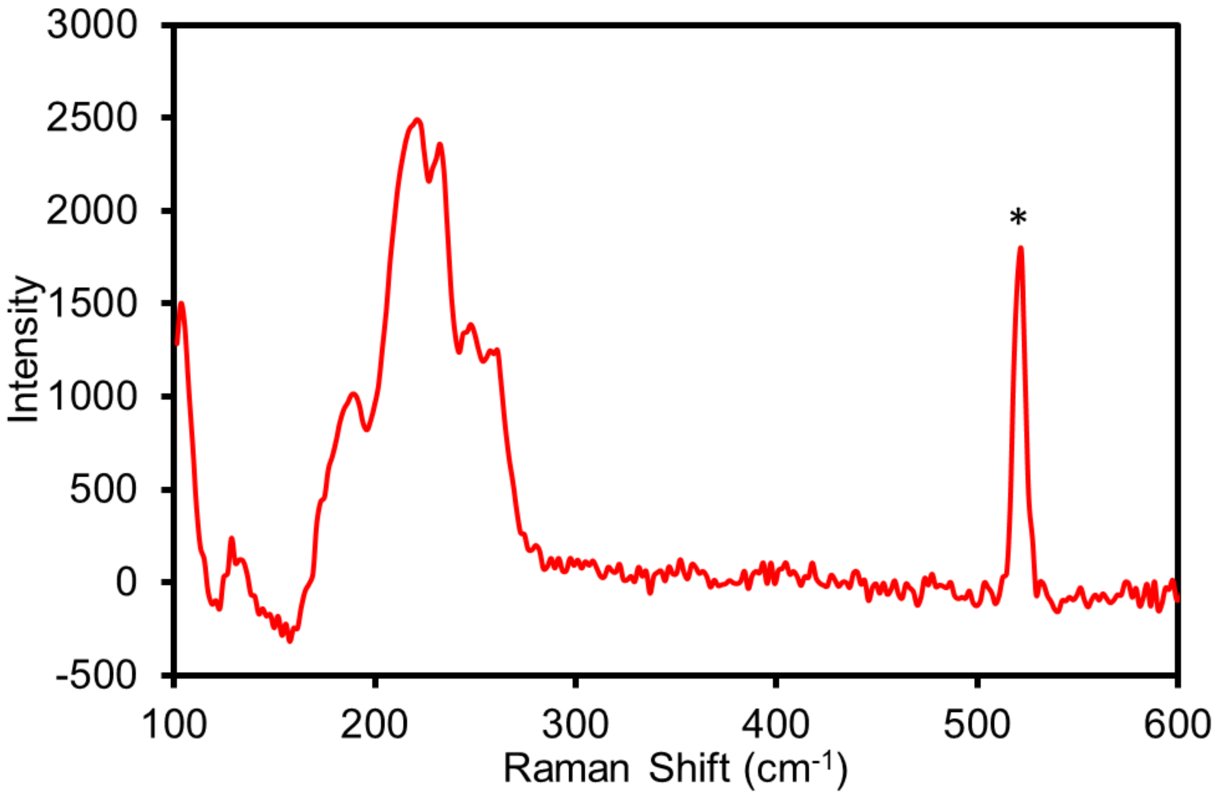

Figure S21. Experimental Raman spectrum of phenylacetate and tributylphosphine ligated InAs clusters using λ_{ex} = 532 nm for exposure. We attribute the signal from 160 – 270 cm^{-1} to the molecular vibrations of the InAs lattice collective. The feature at 522 cm^{-1}, marked by the asterisk, is from the silicon substrate.

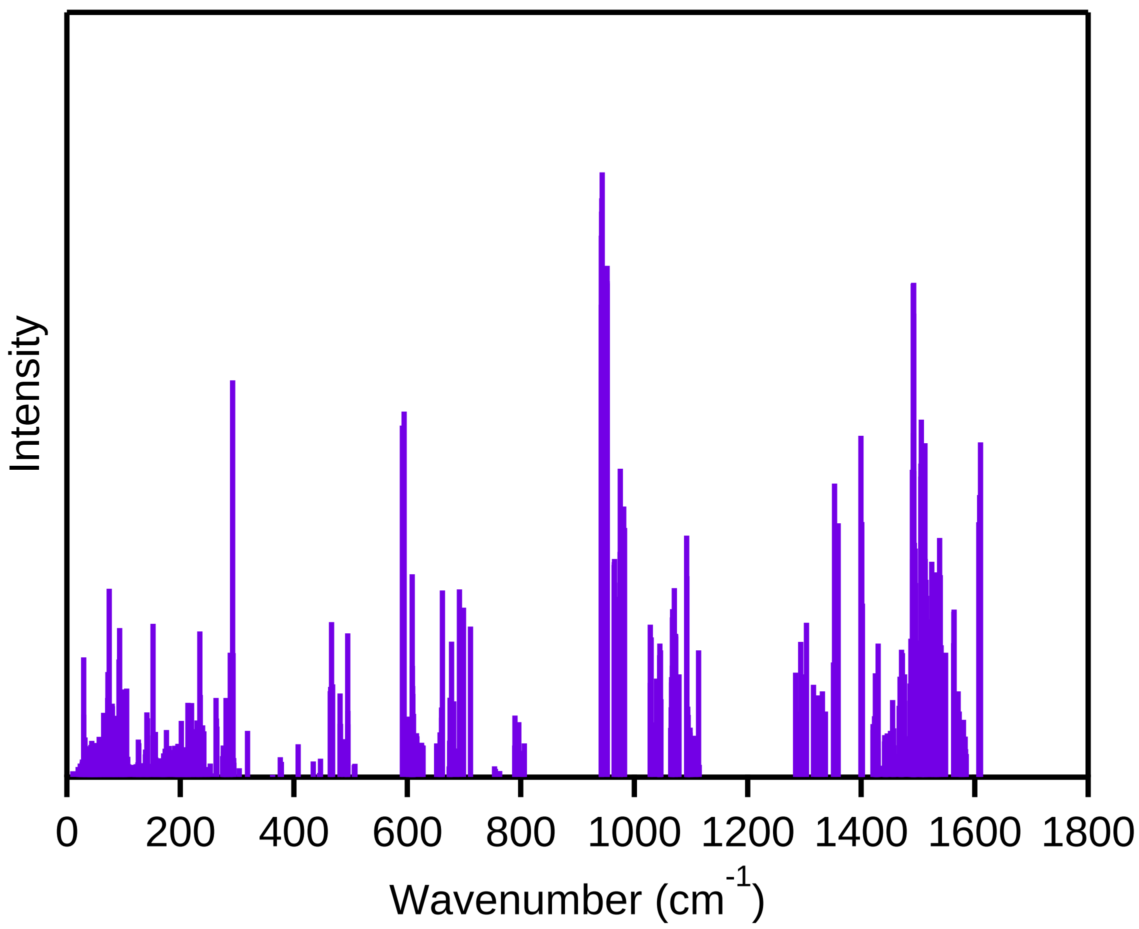

Figure S22. Computational Raman spectrum of the $In_{26}As_{18}$ cluster.

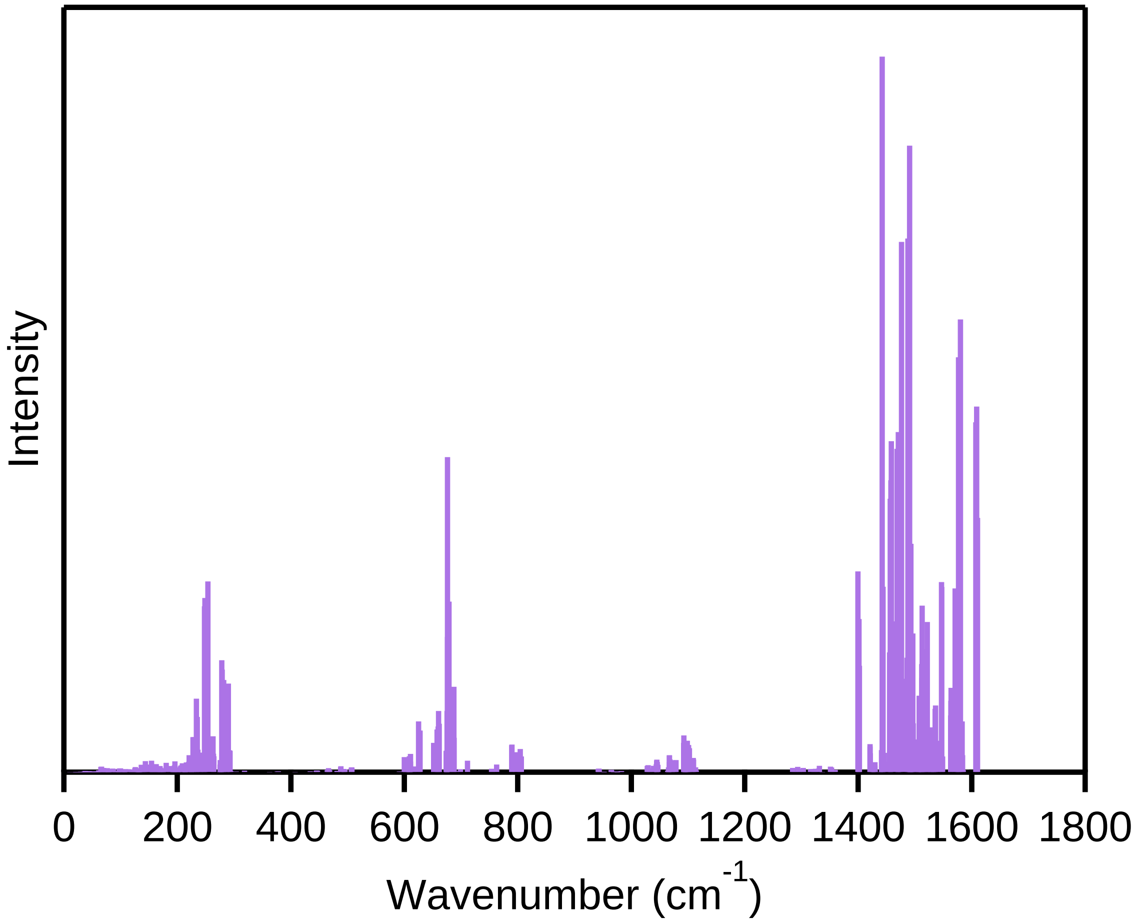

Figure S23. Computational IR spectrum of the $In_{26}As_{18}$ cluster.

References

(1) Gary, D. C.; Cossairt, B. M. Role of Acid in Precursor Conversion During InP Quantum Dot Synthesis. *Chem. Mater.* **2013**, *25* (12), 2463–2469. https://doi.org/10.1021/cm401289j.

(2) Gaussian 16, Revision C.01, Frisch, M. J.; Trucks, G. W.; Schlegel, H. B.; Scuseria, G. E.; Robb, M. A.; Cheeseman, J. R.; Scalmani, G.; Barone, V.; Petersson, G. A.; Nakatsuji, H.; Li, X.; Caricato, M.; Marenich, A. V.; Bloino, J.; Janesko, B. G.; Gomperts, R.; Mennucci, B.; Hratchian, H. P.; Ortiz, J. V.; Izmaylov, A. F.; Sonnenberg, J. L.; Williams-Young, D.; Ding, F.; Lipparini, F.; Egidi, F.; Goings, J.; Peng, B.; Petrone, A.; Henderson, T.; Ranasinghe, D.; Zakrzewski, V. G.; Gao, J.; Rega, N.; Zheng, G.; Liang, W.; Hada, M.; Ehara, M.; Toyota, K.; Fukuda, R.; Hasegawa, J.; Ishida, M.; Nakajima, T.; Honda, Y.; Kitao, O.; Nakai, H.; Vreven, T.; Throssell, K.; Montgomery, J. A., Jr.; Peralta, J. E.; Ogliaro, F.; Bearpark, M. J.; Heyd, J. J.; Brothers, E. N.; Kudin, K. N.; Staroverov, V. N.; Keith, T. A.; Kobayashi, R.; Normand, J.; Raghavachari, K.; Rendell, A. P.; Burant, J. C.; Iyengar, S. S.; Tomasi, J.; Cossi, M.; Millam, J. M.; Klene, M.; Adamo, C.; Cammi, R.; Ochterski, J. W.; Martin, R. L.; Morokuma, K.; Farkas, O.; Foresman, J. B.; Fox, D. J. Gaussian, Inc., Wallingford CT, 2016.

(3) Heyd, J.; Scuseria, G. E. Efficient Hybrid Density Functional Calculations in Solids: Assessment of the Heyd–Scuseria–Ernzerhof Screened Coulomb Hybrid Functional. *The Journal of Chemical Physics* **2004**, *121* (3), 1187–1192. https://doi.org/10.1063/1.1760074.

(4) Heyd, J.; Scuseria, G. E.; Ernzerhof, M. Hybrid Functionals Based on a Screened Coulomb Potential. *The Journal of Chemical Physics* **2003**, *118* (18), 8207–8215. https://doi.org/10.1063/1.1564060.

(5) Vydrov, O. A.; Heyd, J.; Krukau, A. V.; Scuseria, G. E. Importance of Short-Range versus Long-Range Hartree-Fock Exchange for the Performance of Hybrid Density Functionals. *The Journal of Chemical Physics* **2006**, *125* (7), 074106. https://doi.org/10.1063/1.2244560.

(6) Gary, D. C.; Flowers, S. E.; Kaminsky, W.; Petrone, A.; Li, X.; Cossairt, B. M. Single-Crystal and Electronic Structure of a 1.3 Nm Indium Phosphide Nanocluster. *J. Am. Chem. Soc.* **2016**, *138* (5), 1510–1513. https://doi.org/10.1021/jacs.5b13214.

(7) Cho, E.; Jang, H.; Lee, J.; Jang, E. Modeling on the Size Dependent Properties of InP Quantum Dots: A Hybrid Functional Study. *Nanotechnology* **2013**, *24* (21), 215201. https://doi.org/10.1088/0957-4484/24/21/215201.

(8) Leger, J. D.; Friedfeld, M. R.; Beck, R. A.; Gaynor, J. D.; Petrone, A.; Li, X.; Cossairt, B. M.; Khalil, M. Carboxylate Anchors Act as Exciton Reporters in 1.3 Nm Indium Phosphide Nanoclusters. *J. Phys. Chem. Lett.* **2019**, *10* (8), 1833–1839. https://doi.org/10.1021/acs.jpclett.9b00602.

(9) Casida, M. E.; Jamorski, C.; Casida, K. C.; Salahub, D. R. Molecular Excitation Energies to High-Lying Bound States from Time-Dependent Density-Functional Response Theory: Characterization and Correction of the Time-Dependent Local Density Approximation Ionization Threshold. *The Journal of Chemical Physics* **1998**, *108* (11), 4439–4449. https://doi.org/10.1063/1.475855.

(10) Furche, F.; Ahlrichs, R. Adiabatic Time-Dependent Density Functional Methods for Excited State Properties. *The Journal of Chemical Physics* **2002**, *117* (16), 7433–7447. https://doi.org/10.1063/1.1508368.

(11) Stratmann, R. E.; Scuseria, G. E.; Frisch, M. J. An Efficient Implementation of Time-Dependent Density-Functional Theory for the Calculation of Excitation Energies of Large Molecules. *The Journal of Chemical Physics* **1998**, *109* (19), 8218–8224. https://doi.org/10.1063/1.477483.

(12) Bruker (**2007**) APEX2 (Version 2.1-4), SAINT (version 7.34A), SADABS (version 2007/4), BrukerAXS Inc, Madison, Wisconsin, USA.

(13) (a) Altomare A, Burla C, Camalli M, Cascarano G L, Giacovazzo C, Guagliardi A, Moliterni AGG, Polidori G, Spagna R. **(1999)** SIR97: a new tool for crystal structure determination and refinement *Journal of Applied Cryst*allography, ***32,*** 115-119.
(b) Altomare A, Cascarano G L, Giacovazzo C, Guagliardi A. **(1993)** Completion and refinement of crystal structures with SIR 92. *Journal of Applied Cryst*allography, ***26,*** 343-350.

(14) Sheldrick: A short history of SHELX. Acta Cryst. (2008), A64, 112-122

(15) Mackay, S.; Edwards, C.; Henderson, A.; Gilmore, C.; Stewart, N.; Shankland, K.; Donald, A. **(1997)** *MaXus: a computer program for the solution and refinement of crystal structures from diffraction data*. University of Glasgow, Scotland,.

(16) Waasmaier, D.; Kirfel, A. **(1995)** New Analytical Scattering Factor Functions for Free Atoms and Ions. *Acta Crystallographica* A., ***51***, 416-430.

(17) (a) A. Spek, J. Appl. Cryst. (2003), 36, 7-13.
(b) P. van der Sluis & A. L. Spek, Acta Cryst. (1990). A46, 194-201.
(c) Spek, A. L. (2009). Acta. Cryst. D65, 148-155.
